# MALDI-TOF-MS-Based Identification of Monoclonal Murine Anti-SARS-CoV-2 Antibodies within One Hour

**DOI:** 10.3390/antib11020027

**Published:** 2022-04-14

**Authors:** Georg Tscheuschner, Melanie N. Kaiser, Jan Lisec, Denis Beslic, Thilo Muth, Maren Krüger, Hans Werner Mages, Brigitte G. Dorner, Julia Knospe, Jörg A. Schenk, Frank Sellrie, Michael G. Weller

**Affiliations:** 1 Federal Institute for Materials Research and Testing (BAM), Richard-Willstätter-Strasse 11, 12489 Berlin, Germany; georg.tscheuschner@bam.de (G.T.); melliehco@hotmail.de (M.N.K.); jan.lisec@bam.de (J.L.); thilo.muth@bam.de (T.M.); 2 Robert Koch Institute (RKI), Seestraße 10, 13353 Berlin, Germany; beslicd@rki.de (D.B.); kruegerm@rki.de (M.K.); magesh@rki.de (H.W.M.); dornerb@rki.de (B.G.D.); 3 InVivo BioTech Services GmbH/Bruker, Neuendorfstraße 24a, 16761 Hennigsdorf, Germany; julia.knospe@bruker.com; 4 HybroTec GmbH, Am Mühlenberg 11, 14476 Potsdam, Germany; joerg.schenk@hybrotec.com (J.A.S.); frank.sellrie@up-transfer.de (F.S.); 5 UP Transfer GmbH an der Universität Potsdam, Am Neuen Palais 10, 14469 Potsdam, Germany

**Keywords:** SARS-CoV-2 antibody, reproducibility crisis, peptide mass fingerprinting, monoclonal antibody, traceability, identity, antibody identification, antibody light chain, MALDI-TOF-MS

## Abstract

During the SARS-CoV-2 pandemic, many virus-binding monoclonal antibodies have been developed for clinical and diagnostic purposes. This underlines the importance of antibodies as universal bioanalytical reagents. However, little attention is given to the reproducibility crisis that scientific studies are still facing to date. In a recent study, not even half of all research antibodies mentioned in publications could be identified at all. This should spark more efforts in the search for practical solutions for the traceability of antibodies. For this purpose, we used 35 monoclonal antibodies against SARS-CoV-2 to demonstrate how sequence-independent antibody identification can be achieved by simple means applied to the protein. First, we examined the intact and light chain masses of the antibodies relative to the reference material NIST-mAb 8671. Already half of the antibodies could be identified based solely on these two parameters. In addition, we developed two complementary peptide mass fingerprinting methods with MALDI-TOF-MS that can be performed in 60 min and had a combined sequence coverage of over 80%. One method is based on the partial acidic hydrolysis of the protein by 5 mM of sulfuric acid at 99 °C. Furthermore, we established a fast way for a tryptic digest without an alkylation step. We were able to show that the distinction of clones is possible simply by a brief visual comparison of the mass spectra. In this work, two clones originating from the same immunization gave the same fingerprints. Later, a hybridoma sequencing confirmed the sequence identity of these sister clones. In order to automate the spectral comparison for larger libraries of antibodies, we developed the online software ABID 2.0. This open-source software determines the number of matching peptides in the fingerprint spectra. We propose that publications and other documents critically relying on monoclonal antibodies with unknown amino acid sequences should include at least one antibody fingerprint. By fingerprinting an antibody in question, its identity can be confirmed by comparison with a library spectrum at any time and context.

## 1. Introduction

Antibodies belong to the most important biochemical reagents available today to detect, localize, identify and characterize biomolecules as well as their expression and functional status. In addition, therapeutic antibodies and antibody-drug conjugates are extremely valuable for the treatment of severe diseases, such as cancer and infections [1,2,3,4]. In all cases, the unambiguous definition and identification of such reagents or pharmaceuticals are of utmost relevance [5,6]. Therefore, it is more than concerning that in about 50% of all refereed publications, the identity of the used antibodies is not clearly described [7,8,9]. Hence, it was repeatedly demanded that only recombinant antibodies with full disclosure of their sequence should be used in scientific work [10,11,12,13]. Unfortunately, this approach seems to be unfeasible in the foreseeable future due to different reasons, such as cost and the protection of intellectual property [14,15]. Nevertheless, the independent identification of antibodies of all kinds seems to be indispensable to ensure the reproducibility of any research work performed with antibodies. However, many antibody resellers rename antibodies and do not disclose their original clone names or respective original publications [16,17]. Therefore, the purchase of an antibody might not ensure a clear reference to original work, and to make it even worse, many scientists are not even aware that their Materials section in publications is not satisfactory to refer to a specific antibody (clone) [18,19,20,21]. If the corresponding hybridoma cell is accessible, the sequencing of the respective antibody does not seem to be complicated. However, this situation is a rare exception as the sequence information is usually not disclosed to the public due to the intellectual property issues as mentioned above. In most cases, only the antibody itself is available in limited amounts of a few hundred micrograms. In this situation, a protein sequence might hardly be obtained, or the cost and effort of such protein sequencing may be prohibitive.

Conventionally, sequence-independent identification of antibodies on the protein level is performed by peptide mapping [22,23,24]. Peptide maps are mostly generated by tryptic cleavage followed by LC separation and UV-vis absorbance and/or MS detection. The main advantage of this method lies in the quantity of information gained about the sample. Consequently, even often unwanted modifications such as deamidation, oxidation, or other degradation products can be detected, making LC-MS-based identification a valuable tool for the quality control of monoclonal and recombinant antibodies. Nevertheless, as a tool for the fast and straightforward identification of antibodies, these protocols for the generation of peptide maps fail to convince. Finally, the lack of independent sequence information makes these data insufficient to identify an antibody.

Therefore, it is understandable that these protocols are rarely (if at all) used for antibody identification or traceability purposes. In order to avoid these complex and lengthy protocols, we tried to make our methods as simple and as fast as possible. In a first publication, we have shown the potential of MALDI-TOF-MS fingerprinting [25]. In contrast to LC-ESI-MS, MALDI-TOF-MS allows for a much quicker analysis without the need for a chromatographic step, even for complex peptide samples [26]. With the approach we present in this work, identifying an antibody in a database would be possible in less than 60 min by performing MALDI-TOF-MS of a digested sample based on 10 µg of protein.

As a practical example, we examined 35 different antibodies against SARS-CoV-2 antigens. Due to the high number of antibodies raised against SARS-CoV2-2 in recent times, this set of samples seemed appropriate to exemplify the traceability of these antibodies. A multistep approach was tested to determine how much information would be needed to identify a specific antibody. First of all, we examined the molecular mass of the antibodies and their light chain. As the next step, the cleavage of the antibody by diluted acid [27,28,29,30,31,32] and by tryptic digest were performed. The resulting peptide mass fingerprints [33,34] can be compared by visual inspection to determine whether two clones are identical or not. In the case of visually similar fingerprints, our software, ABID 2.0 (Antibody Identifier, https://bam.de/ABID, accessed on 5 April 2022 ), may determine the number of overlapping peaks and facilitate the decision. Furthermore, ABID 2.0 allows the creation of antibody fingerprint libraries, which can be used to link the identity of an antibody to scientific work without much effort and dependence on more or less arbitrary names such as clone designations. As a proof-of-concept, we also managed to show that the approach can even determine antibody subclasses.

## 2. Materials and Methods

The NIST monoclonal antibody reference material 8671 (NIST-mAb 8671) [34,35] was used to optimize all methods and as a relative mass calibrator. The panel of 35 SARS-CoV-2 antibodies used in this study is shown in Table 1.

All antibodies were delivered in PBS. They were produced with different antigens: Spike glycoprotein S1 (S1), receptor-binding domain (RBD), or nucleocapsid protein (N).

Other materials and reagents were obtained as follows: Acetonitrile 99.95% (ACN, 2697) was purchased from Th. Geyer GmbH & Co. KG (Renningen, Germany). Bruker Peptide Calibration Standard II (8222570) was purchased from Bruker (Billerica, MA, USA). Tris(2-carboxyethyl)phosphine hydrochloride 98% (TCEP, 580560) was obtained from Calbiochem (San Diego, CA, USA). 2,5-Dihydroxyacetophenone (DHAP, A12185) was purchased from Alfa Aesar (Haverhill, MA, USA). Sulfuric acid (5 mM, 28-6020), sinapinic acid > 98% (D7927), tris-(hydroxymethyl)-aminomethane hydrochloride (T3253), tris(hydroxymethyl)aminomethane (T1503), and recombinant trypsin (3708985001) were purchased from Sigma-Aldrich (St. Louis, MO, USA). Trifluoroacetic acid 99.5% (TFA, 85183), Pierce C18 tips (10 µL, 87784), and Zeba Micro Desalting Columns 7K MWCO (89877) were obtained from Thermo Fisher (Waltham, MA, USA). Lab water was taken from a Milli-Q water purification system (Millipore, Bedford, MA, USA) with a resistivity of >18.2 Ω and TOC value of <5 ppb.

### 2.1. Determination of Intact Masses (IgG)

Antibodies with a concentration above 0.5 mg/mL were diluted 1:10 in purified water. From this dilution, 1 µL was spotted on a MALDI target plate. An amount of 1 µL of sinapinic acid matrix solution (10 mg/mL with 30% acetonitrile, 69.9% purified water, 0.1% trifluoroacetic acid) was added, and after mixing, the droplet was left to dry. Antibodies with a concentration below 0.5 mg/mL or antibodies that did not give satisfactory spectra even at higher concentrations were desalted using Zeba Spin 7K MWCO size-exclusion desalting columns (75 µL) according to the manufacturer’s protocol. An amount of 1 µL of the eluted solution was pipetted on a target spot on the MALDI plate, and 1 µL of sinapinic acid matrix solution was added. After mixing, the droplet was left to dry. For every antibody, three sample spots were prepared from the same solution. The spots on the MALDI target were chosen in a way that all antibodies were in close proximity to the reference antibody NIST-mAb 8671. MALDI measurements were performed on a Bruker Autoflex maX in linear mode. 5000 laser shots were accumulated to obtain a spectrum. For each antibody spectrum, the peak of the doubly charged species at around 75.000 Da was smoothed in Origin (Version 2021, 64-bit) with a Savitzky-Golay Filter (50 points), and the maximum was determined by manual peak picking of the smoothed curve.

### 2.2. Determination of Light Chain Masses (IgG)

In a 0.2 mL PCR tube, the volume of the antibody solution (usually around 10 µL) corresponding to 10 µg of the antibody was mixed with 5 µL 1 M tris(hydroxymethyl)aminomethane buffer pH 7.8 (2.2127 g Tris-HCl + 0.7877 g Tris-base + 20 mL purified water) and a tris(2-carboxyethyl)phosphine solution (TCEP final concentration: 0.1 mM). The solution was incubated for 15 min at room temperature and 950 rpm on an Eppendorf Thermo Mixer with a SmartBlock PCR 96. Afterward, the solution was desalted using Zeba Spin 7K MWCO size-exclusion desalting columns (75 µL) according to the manufacturer’s protocol. An amount of 1 µL of the eluted solution was pipetted on a target spot on the MALDI plate, and 1 µL of sinapinic acid matrix solution was added. After mixing, the droplet was left to dry. For every antibody, three sample spots were prepared from the same solution. The spots on the MALDI target were chosen in a way that all antibodies were in close proximity to the reference antibody NIST-mAb 8671. MALDI measurements were performed on a Bruker Autoflex maX in linear mode. A total of 5000 laser shots were accumulated to obtain a spectrum. For each spectrum of the reduced antibody, the peak of the singly charged species at around 23.000 Da was smoothed in Origin (Version 2021, 64-bit) with a Savitzky-Golay Filter (50 points), and the maximum was determined by manual peak picking of the smoothed curve.

### 2.3. Cleavage with Diluted Sulfuric Acid

In a 0.2 mL PCR tube, the volume of the antibody solution (usually around 10 µL), corresponding to 10 µg of the antibody, was mixed with a final TCEP concentration of 0.1 mM and sulfuric acid (5 mM) so that the final volume of the solution was 150 µL. Antibody cleavage was carried out for 30 min at 99 °C and 950 rpm on an Eppendorf Thermo Mixer with a SmartBlock PCR 96. No alkylation step was performed. Afterward, peptides were enriched and washed with Pierce C18 tips (10 µL) according to the manufacturer’s protocol. Peptides were eluted from the peptide tip with 2 µL of 2,5-dihydroxyacetophenone (DHAP) MALDI matrix solution (10 mg/mL, 69.9% purified water, 30% ACN, 0.1% TFA) directly on a spot of the MALDI target. MALDI measurements were performed on a Bruker Autoflex maX in reflector mode. The instrument was calibrated with the Bruker Peptide Calibration Standard II using DHAP as the MALDI matrix. A total of 5000 laser shots were accumulated to obtain a fingerprint spectrum.

### 2.4. Enzymatic Cleavage with Trypsin

In a 0.2 mL PCR tube, the volume of the antibody solution (usually around 10 µL) corresponding to 10 µg of the antibody was mixed with a final TCEP concentration of 0.1 mM and 0.1 M Tris buffer (pH 7.8) so that the final volume of the solution was 50 µL. Tris buffer (pH 7.8) was prepared in the following way: 0.7877 g tris(hydroxymethyl)aminomethane and 2.127 g tris(hydroxymethyl)aminomethane hydrochloride were dissolved in 200 mL purified water, and the pH was adjusted to 7.8 by adding a few drops of 1 M HCl if necessary. Denaturation of the antibody and disulfide cleavage was carried out by incubating the solution for 15 min at 99 °C and 950 rpm on an Eppendorf Thermo Mixer with a SmartBlock PCR 96. No alkylation step was performed. Afterward, the solution was cooled down to 55 °C, and trypsin was added in a trypsin-to-antibody mass ratio of 1:120. The digestion was allowed to proceed for another 15 min at 55 °C before 10 µL of a 0.1% TFA solution was added. Peptide purification and MALDI measurements were performed as described in Section 2.3.

### 2.5. Software-Assisted Antibody Identification with ABID 2.0

ABID 2.0 is a web-based software tool that can be accessed with the following link: https://bam.de/ABID (accessed on 5 April 2022). It can be used online and does not need to be downloaded and installed on a local device. For the majority of functions and parameters on the graphical user interface, explanations are provided on mouse hovering. For further questions regarding the software, please contact one of the authors. The complete R code is publicly available here: https://github.com/BAMresearch/ABID (accessed on 8 April 2022). Raw data of all spectra are accessible on Zenodo (https://doi.org/10.5281/zenodo.6375803, accessed on 5 April 2022).

In a previous work [25], we published the java tool ABID. The idea of the 2.0 version described here is similar. Both tools allow the creation of fingerprint libraries and use peak detection algorithms to generate peak lists that can be matched against a sample spectrum to identify an antibody. ABID 2.0 has several advantages over ABID, which are discussed in Section 3.4. For subclass determination, antibody sequences were obtained from the IMGT/mAb-DB and UniProt databases. Peptide masses were generated from CH2-CH3 regions using MS-Digest by the Protein Prospector package (https://prospector.ucsf.edu/prospector/cgi-bin/msform.cgi?form=msdigest, accessed on 5 April 2022). Trypsin and Asp-N/Asp-C were chosen as in silico digestion methods, respectively. By comparison of a sample spectrum with the library entries of subclass-specific peptides, ABID 2.0 determines the best subclass match based on the number of matching peptides.

## 3. Results

### 3.1. Determination of Intact Masses

**Figure 1:**
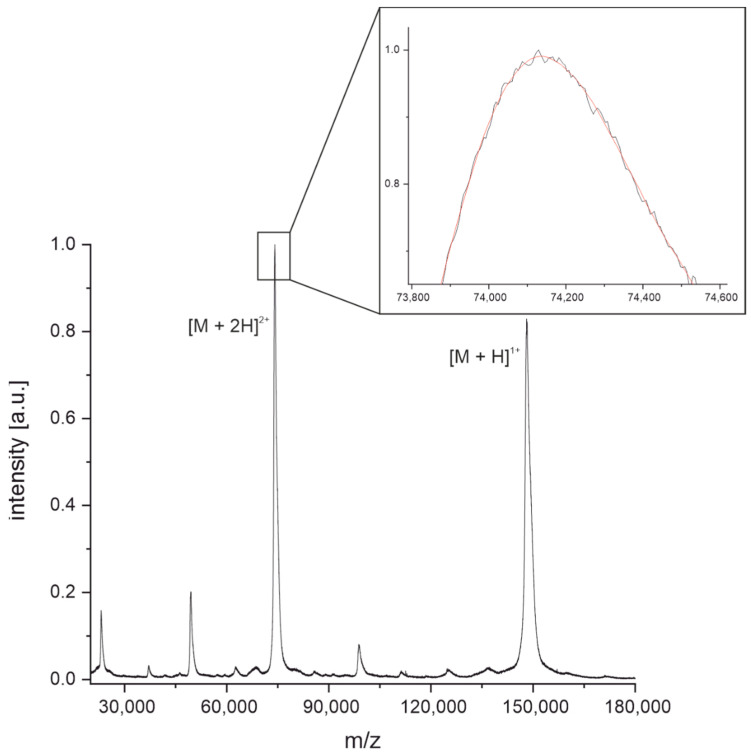
MALDI-TOF mass spectrum of intact NIST-mAb 8671. Intact mass was determined by smoothing the peak of the doubly charged species using a Savitzky-Golay filter with 50 points (inset, red curve) followed by manual peak picking of the maximum of the curve at m/z. The intact mass M was then calculated from the m/z value of the doubly charged species [M + 2H]^2+^.

Intact masses of antibodies were determined relative to the mass of the monoclonal reference antibody NIST-mAb 8671. The purpose of this approach is the traceability of the developed method. Calibration of MALDI-TOF-MS instruments in mass ranges of around 150 kDa tends to be error-prone due to broad and asymmetric peak shapes. Smoothing of the peak maximum, as shown in Figure 1 for the doubly charged species of the NIST-mAb 8671, allows for a more reproducible way to determine the peak maximum. Smoothing (Figure 1, inset, red curve) was done with a Savitzky-Golay filter with 50 points for the entire peak. The intact mass M of the NIST-mAb 8671 was then calculated from the doubly charged species [M + 2H]^2+^. Triplicates were measured to determine a confidence interval of 95%. All antibodies used for this work were measured in the same way as the NIST-mAb 8671.

**Figure 2:**
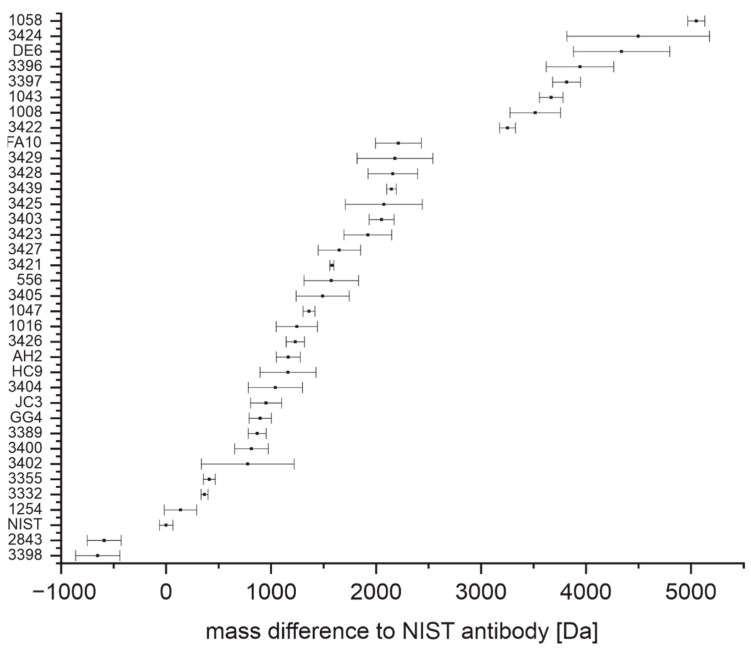
Intact masses of anti-SARS-CoV-2 antibodies relative to NIST-mAb 8671 given by a confidence level of 95%.

In Figure 2, all antibodies used in this work and their mass differences with a confidence level of 95% are plotted. Based on their intact masses, distinguishing between certain antibodies is possible. However, many antibodies do not have a unique mass, which means that their confidence intervals overlap with confidence intervals of one or more of the other antibodies.

### 3.2. Determination of Light Chain Masses

**Figure 3:**
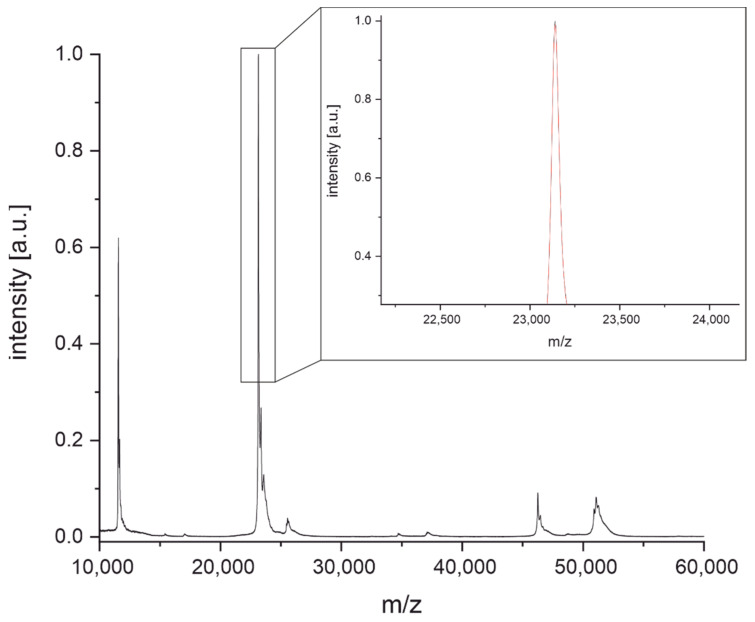
MALDI-TOF mass spectrum of NIST-mAb 8671 after cleavage of intermolecular disulfide bonds. The mass of the light chain was determined using a Savitzky-Golay filter with 50 points (inset, red curve) followed by manual peak picking of the maximum.

Light chain masses of antibodies were also measured relative to the light chain mass of the reference antibody NIST-mAb 8671. This also serves the purpose of traceability. Intermolecular disulfide bonds of NIST-mAb 8671 were reduced by incubation with tris(2-carboxyethyl)phosphine (TCEP) for 15 min at room temperature. Afterward, the solution was purified from excess TCEP and salts using a Zeba Spin Micro Desalting Column with a molecular weight cut-off of 7 kDa. The mass spectrum in Figure 3 shows the heavy chain at around 51 kDa, the light chain dimer at around 46 kDa, the light chain at around 23 kDa, and their doubly charged species at around 12.5 kDa. The peak at 23 kDa has a high intensity and is much sharper compared to the peak of the intact antibody shown in Figure 1. Therefore, the precision of the mass determination can be expected to be much higher.

**Figure 4:**
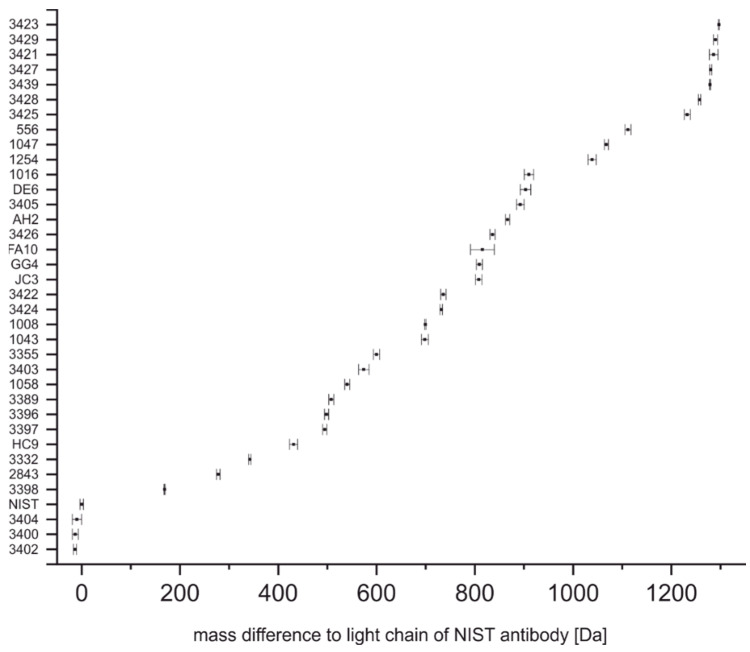
Light chain masses of anti-SARS-CoV-2 antibodies relative to NIST-mAb 8671 given with a confidence level of 95%.

Triplicates of all antibodies used in this study were measured. The comparison of all light chain masses relative to the NIST-mAb 8671 can be seen in Figure 4, indicating a confidence level of 95%. As expected, the overlap of the confidence levels is much smaller when comparing light chain masses only. Several antibodies (17 out of 36) were unique in this set of antibodies solely based on their light chain mass. However, some antibodies still seem to have overlapping light chain masses.

**Figure 5:**
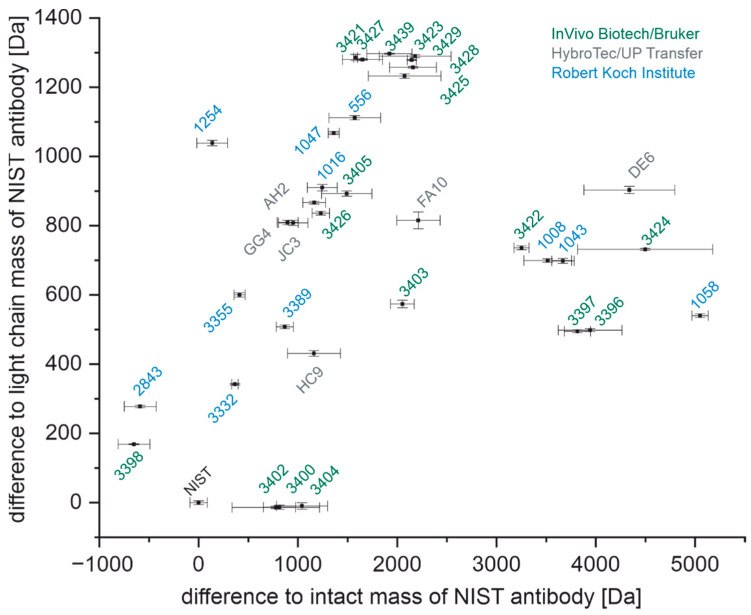
Light chain and intact masses of all antibodies relative to the NIST antibody, including their respective confidence intervals. The remaining non-unique antibodies are shown in Figure S1. Antibodies raised from immunization by InVivo Biotech/Bruker are colored in green, antibodies raised from immunization by HybroTec/UP Transfer are colored in grey, and antibodies raised by the Robert Koch Institute are colored in blue.

Figure 5 shows all antibodies with their confidence intervals for their light chain masses plotted against their intact masses. A total of 21 out of 36 antibodies do not have overlapping confidence intervals for both their light chain and intact masses and can therefore be unambiguously identified this way. The combination of the light chain and intact masses revealed four new unique antibodies (FA10, DE6, 3422, 3424) that could not have been distinguished by one of the methods alone.

Interestingly, almost all antibodies with overlapping confidence intervals originate from clones of the same immunization (see Figure S1). This could be due to high sequence similarity up to complete identity. Only the antibodies 1016 and 3405 with overlapping confidence intervals originate from different immunizations.

### 3.3. Peptide Mass Fingerprinting

#### 3.3.1. Cleavage with Diluted Sulfuric Acid

In order to examine the differences of antibodies down to the amino acid level, a peptide mass fingerprinting method was developed. Previously, we demonstrated the effectiveness of MALDI-TOF-MS in antibody fingerprinting to prove antibody identity [25].

**Figure 6:**
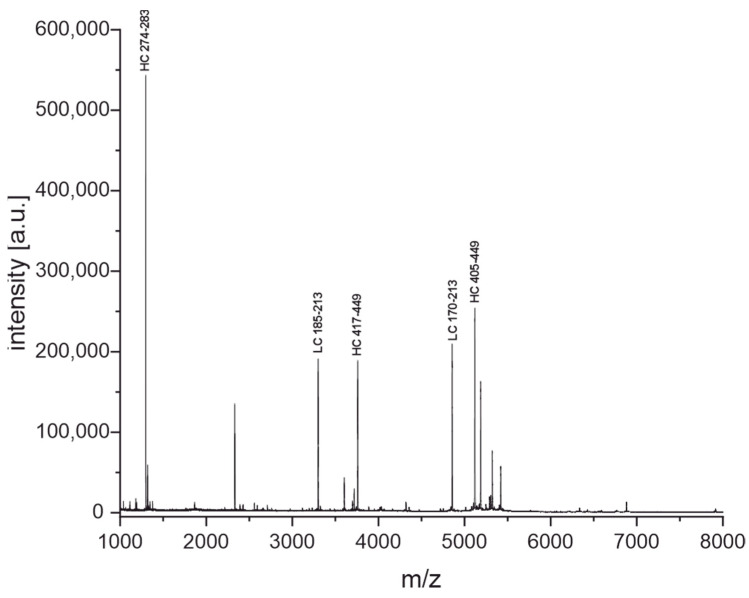
MALDI-TOF fingerprint spectrum of NIST-mAb 8671 after 30 min of incubation with diluted sulfuric acid and TCEP at 99 °C. The five most intense peptide peaks are assigned to the sequence of the NIST antibody in the spectrum (HC heavy chain, LC light chain). Sequence assignment was possible for most peaks, including variable and hypervariable regions (see Figure S2). The sequence coverage was around 61%.

Here, we present an improved and extended version of this protocol. The NIST-mAb 8671 [35,36] was used for the development of the method. Ten micrograms of the antibody were incubated with TCEP and diluted sulfuric acid at 99 °C for 30 min. Alkylation was not necessary in this context. Afterward, the peptides were purified using Thermo Pierce C18 tips (10 µL). Elution was performed with 2 µL of the MALDI matrix solution (2,5-dihydroxyacetophenone, DHAP) directly on the MALDI plate. The whole experiment can be finished in under 60 min. This is a significant advantage over commonly used peptide fingerprinting protocols for antibodies that tend to be rather long with several clean-up steps [37]. The fingerprint spectrum of NIST-mAb 8671 obtained after the cleavage with diluted sulfuric acid is shown in Figure 6. The mass spectrum is rich in peptide information and has a good signal-to-noise ratio. The five most intense peptide peaks are exemplarily assigned to the amino acid sequence of the NIST antibody. The remaining assignments can be found in the SI (Figure S2). The sequence coverage for the NIST antibody is around 61%. However, it has to be stressed that the determination of the sequence coverage is the exception because sequence information is not available in most cases.

For the development of the cleavage method, we tested several weak or diluted acids for selective cleavage at aspartic residues (SI, Figure S3). Diluted sulfuric acid gave the best results regarding peak numbers and signal-to-noise ratio. The use of sulfuric acid also avoids unwanted peptide modifications such as formylation or acetylation commonly seen in the products derived from fingerprinting of proteins performed with formic or acetic acid [38,39]. To the best of our knowledge, this is the first time that diluted sulfuric acid has been reported as a cleavage agent for the generation of peptide mass fingerprints. However, it has to be noted that the cleavage mechanism can be assumed to be the same as with diluted organic acids that have previously been reported for the cleavage of proteins [27,28,29,30,31,32].

Several other parameters were optimized. For example, it is advisable to use 2,5-DHAP as a MALDI matrix in order to generate good fingerprints. The comparison of different MALDI matrices can be found in the SI (Figure S4).

#### 3.3.2. Enzymatic Cleavage with Trypsin

**Figure 7:**
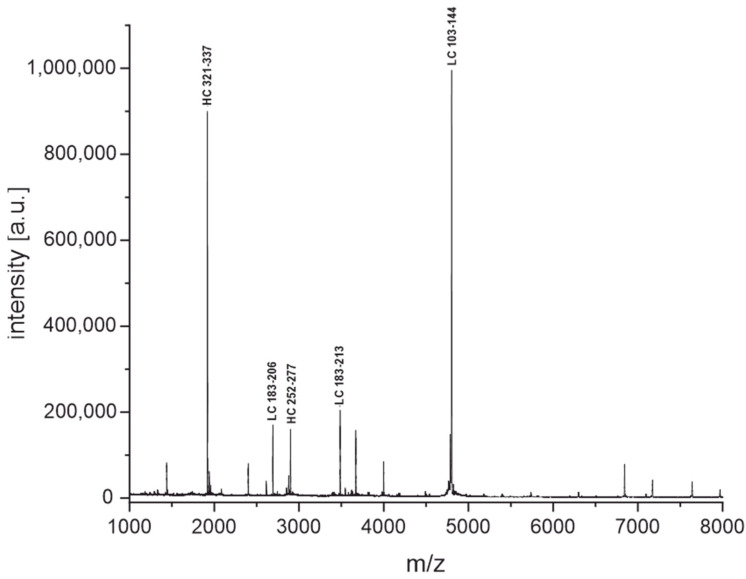
MALDI-TOF fingerprint spectrum of NIST-mAb 8671 after 15 min of incubation with TCEP at 99 °C, followed by 15 min of incubation with trypsin at 55 °C. The five most intense peptide peaks are assigned to the sequence of the NIST antibody in the spectrum (HC heavy chain, LC light chain). Sequence assignment was possible for most peaks (see SI, Figure S5). The sequence coverage was around 65%.

Another fingerprinting method with a complementary cleavage mechanism was developed to obtain additional peptides useful for identification. This protocol is based on the well-established enzymatic digestion with trypsin. However, in contrast to conventional tryptic digestion protocols, the denaturation of the antibody is achieved with heat rather than with chaotropic reagents such as guanidinium hydrochloride. The main advantage is that a sample clean-up prior to the addition of trypsin is not necessary [40]. After incubation of the antibody in tris buffer with 0.1 mM TCEP at 99 °C for 15 min, the sample was cooled down to 55 °C, and trypsin was added. Afterward, the peptides were purified as described in Section 3.3.1. The resulting fingerprint spectrum is shown in Figure 7. This technique also seems to produce a large amount of peptide-level information of the antibody. The five most abundant peptides were again assigned to the sequence of the NIST antibody; remaining assignments can be found in the SI (Figure S5). The sequence coverage for the NIST antibody was around 65%. It is important to note that alkylation did not improve the sequence coverage. Figure S6 in the supplementary information shows sequence coverage with and without an alkylating step. We could show that the combined sequence coverage from the fingerprints generated by acidic cleavage and tryptic digestion is around 82% (SI, Figure S7), with five out of six complementarity-determining regions (CDR) covered.

**Figure 8:**
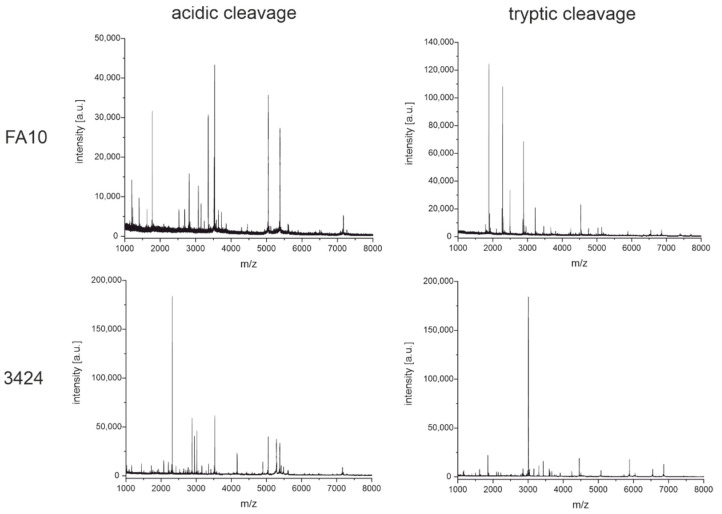
MALDI-TOF fingerprint spectra of the monoclonal antibodies FA10 and 3424 were obtained from cleavage with diluted sulfuric acid for 30 min at 99 °C and tryptic digestion for 15 min at 55 °C. Antibodies FA10 and 3424 originated from different immunizations and have different light chain and intact masses.

These fingerprinting techniques were used to elucidate the characteristics of the antibodies shown in Figure 5. First, we intended to demonstrate how to distinguish antibodies that do not share common characteristics, such as the same light chain or intact masses. Figure 8 shows the fingerprints of two antibodies, FA10 and 3424, where this is the case. It is rather obvious that the fingerprints are not matching and nicely complement the results from the light chain and intact mass measurements. We want to emphasize that the overwhelming majority of antibodies used in this study produce obviously unique fingerprints, especially when comparing clones from different immunizations where slight permutations of the same amino acid sequence should be rare. Therefore, we believe this to be a powerful tool to identify and distinguish between antibodies targeting either the same or different antigens. The fingerprints (acidic and tryptic cleavage) for all 35 SARS-CoV-2 antibodies used in this study can be found as figures in the supplementary information, and as raw data files on a public repository (https://doi.org/10.5281/zenodo.6375803, accessed on 5 April 2022) or in the software tool ABID 2.0 (https://bam.de/ABID, accessed on 5 April 2022, as a virtual library with processing options).

**Figure 9:**
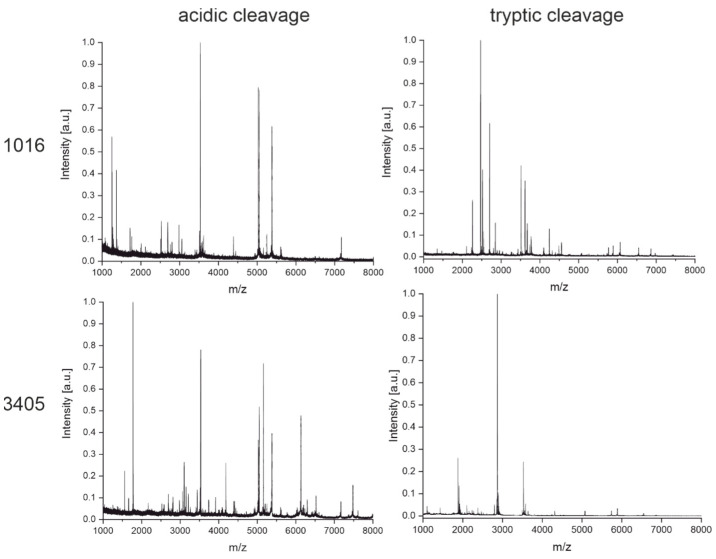
MALDI-TOF fingerprint spectra of the monoclonal antibodies 1016 and 3405 were obtained from cleavage with diluted sulfuric acid for 30 min at 99 °C and tryptic digestion for 15 min at 55 °C. Antibodies 1016 and 3405 originated from different immunizations but have similar light chain and intact masses. However, some obvious differences showed up in the fingerprints based on acidic cleavage and tryptic digest.

In a subsequent comparison, antibodies 1016 and 3405 were examined. They seem to be rather similar based on their intact and light chain masses but originate from different immunizations. Hence, it can be ruled out that they are sister clones. For both antibodies, two fingerprinting techniques with diluted sulfuric acid and trypsin as cleavage agents were performed (Figure 9). Indeed, the spectra show certain similarities. Especially the cleavage with diluted sulfuric acid appears to produce many similar peptides for both antibodies. On the other hand, some peptides seem to be unique for one of the antibodies, for example, the peaks at around 1500 Da or 6000 Da. In addition, tryptic digestion produces rather different fingerprints. The similarities of both antibody fingerprint spectra can mainly be explained by the same subclass (IgG1). Peptides derived from the Fc-region would then be expected to have the same masses or m/z, respectively. In this case, the question of the antibody’s identity might not be immediately answered by visual inspection alone. The comparison is facilitated by the software-assisted analysis of the fingerprints with our tool ABID 2.0, explained in Section 3.4 in more detail.

In Figure 5, another example is shown with the antibody pair 1008 and 1043. Both target the same antigen (the RBD of the SARS-CoV-2 spike protein). These two antibodies seem to have very similar, if not the same, intact and light chain mass. They also originate from the same immunization. In such cases, when the screening of hybridoma clones gives several positive hits, it would be helpful to have a fast and cheap method to determine the uniqueness of the antibodies before the next steps. Growing and recloning several clones can be costly and time-consuming, and it may be found later that two or several clones have the same sequence.

**Figure 10:**
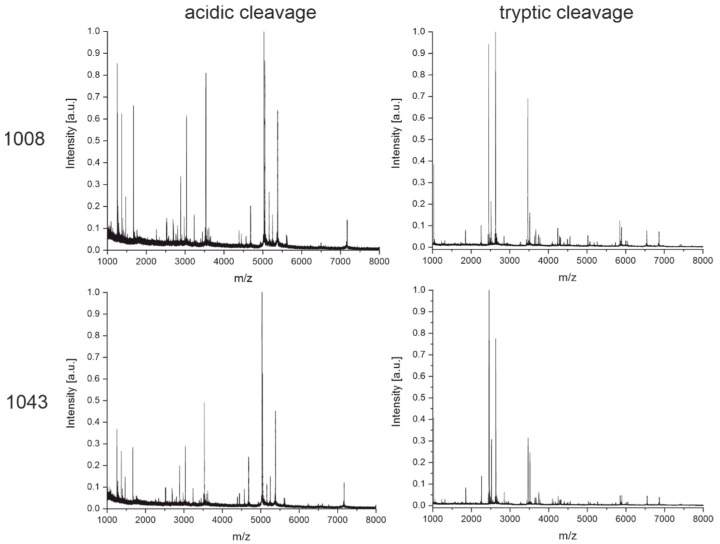
MALDI-TOF fingerprint spectra of the monoclonal antibodies 1008 and 1043 were obtained from cleavage with diluted sulfuric acid for 30 min at 99 °C and tryptic digestion for 15 min at 55 °C. Antibodies 1008 and 1043 originated from the same immunization and turned out to be identical after a later hybridoma sequencing. The similarity is evident even by a brief visual inspection.

In Figure 10, the fingerprints of both cleavage techniques for antibodies 1008 and 1043 show very similar patterns. Based on the fingerprints, it can be suspected that both clones are at least sister clones of a very similar sequence. Later, it could be confirmed by hybridoma sequencing that both clones are indeed identical (Krüger et al., manuscript in preparation). This case shows the practical usefulness of the fingerprinting approach. For the software-assisted analysis of this case, see Section 3.4.

### 3.4. Software-Assisted Antibody Identification with ABID 2.0

In some cases, a visual comparison of two fingerprint spectra may not be sufficient to identify a difference between similar clones. For these cases, a software-assisted evaluation can be helpful. Previously, we reported the confirmation of antibody identity with the help of our java tool ABID (Antibody Identifier) [25].

Here, we offer an improved version of this tool, ABID 2.0. The software uses a peak detection algorithm and compares the peptide masses of a sample spectrum with all spectra from a library. The best match based on the number of identical peptides can be expected to be the same or at least a very similar antibody if no other antibodies in the library have a similar number of matching peptides. ABID 2.0 can store a database of fingerprints which can be used for spectral comparison with a sample spectrum. In its current version (0.61), the pre-implemented default database contains all the antibodies shown in Table 1. For each antibody, fingerprints of the acidic cleavage and the tryptic digestion are stored.

**Figure 11:**
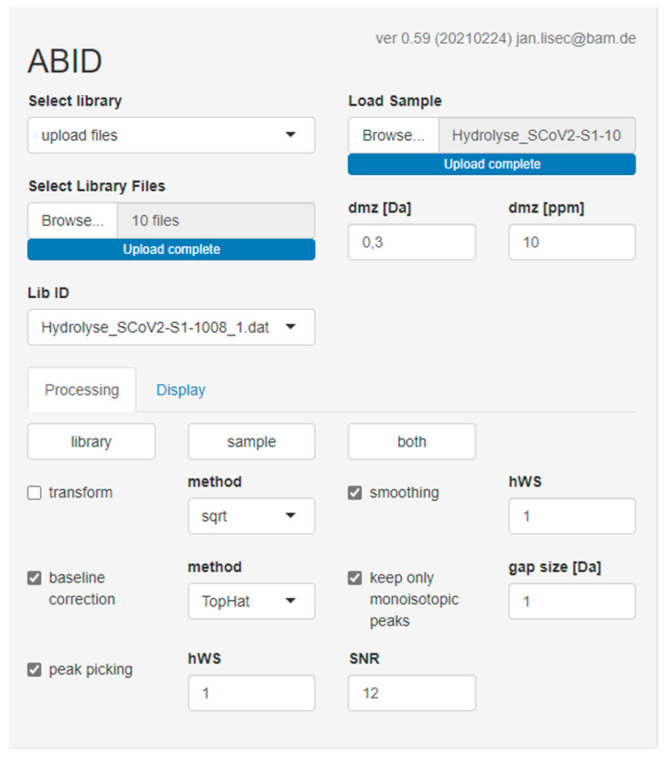
Screenshot of the graphical user interface of the ABID 2.0 software. Antibody fingerprints are stored or uploaded and processed to generate a spectra library. This library can then be compared to an independent spectrum (“Load Sample”). Several processing parameters can be changed to improve the matching, for example, for noisy spectra.

Additionally, the library contains the fingerprints of the NIST reference antibody 8671-mAb. This spectral library can be expanded by the addition of fingerprints of other antibodies and their metadata. The authors would be glad to receive antibody fingerprints from interested readers and add them to the library. The search function on the graphical user interface allows the user to filter the library. This is useful if only antibodies against a certain antigen are compared to the sample spectrum. For example, typing “RBD” in the search bar will filter out all antibodies that do not target the RBD. Contrary to the default database, the user now has the option to upload a temporary library of his own. In Figure 11, a screenshot of the user interface of ABID 2.0 is shown. Choosing “upload files” and clicking “browse” will ask the user to upload the raw data. This can be used to check if an individual sample spectrum (“load sample”) is identical to one of the spectra in the temporary library. After uploading the sample spectrum and the library spectra, several processing parameters can be chosen. For example, it is possible to alter the mass tolerance (dmz) to be in line with the quality of calibration and the resolution of the mass spectrometer. Furthermore, library and sample spectra are optionally smoothed and baseline corrected for noisy measurements. Additionally, several peak-picking parameters may be optimized if required. However, in our experience, the chosen parameters shown in Figure 11 will give satisfactory results for the majority of data.

**Figure 12:**
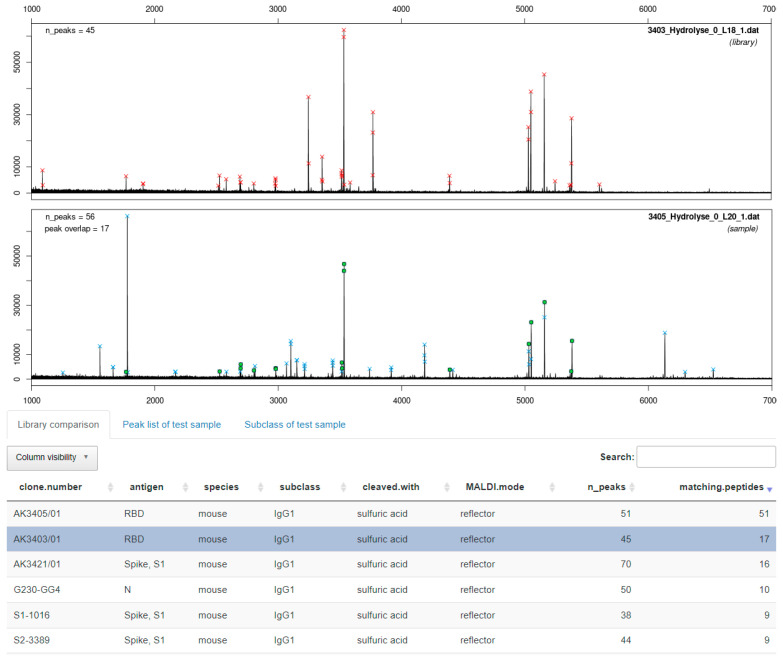
Screenshot of the graphical user interface of the ABID 2.0 software. The antibody 3405 (bottom spectrum, fingerprint with acidic cleavage) is matched against a library of 36 antibodies containing the clone 1016. The same spectra are also compared in Figure 9. The software finds 51 matching peptides (100%) with the library spectrum of the sample spectrum (first row). Seventeen matching peptides are found for clone 3403 (highlighted in blue). However, it also finds a comparable number of peptides with other clones in the library, meaning no clone in this library is identical to 3405.

In the following, we will demonstrate how ABID 2.0 can be used to aid the spectral comparison of fingerprints. For this purpose, we used the pre-implemented default database of all SARS-CoV-2 antibodies used in this study as a reference library. Next, a sample spectrum is loaded and compared to the library. As a first example, two clones, which are undoubtedly different, but whose fingerprints appear to be rather similar, are checked. The fingerprints generated from the acidic cleavage of clones 1016 and 3405 shown in Figure 9 are chosen. The fingerprint of 3405 was used as the sample spectrum (“Load Sample”) that was compared to the default library of 36 clones (35 SARS-CoV-2 antibodies and the NIST-mAb 8671). Figure 12 shows the results of the software delivered after comparing the sample spectrum with the library. In the bottom panel, the fingerprints of the antibody in the library (including all the metadata) are sorted by the number of matching peptides (last column) with the sample spectrum. N_Peaks is the number of peaks that the software detects in total for a single spectrum, given the chosen processing parameters.

Consequently, the sample spectrum (seen in the middle panel) has 100% matching peptides with the first entry of the library, clone 3405, because this spectrum is already present in the library. The second-best match (highlighted in blue) is clone 3403 with 17 matching peptides. After examination of the top library entries, it is evident that no precise match to the antibody 3405 can be found, indicating that this antibody is unique in the sample set. The seemingly similar clone 1016 (see Figure 5 and Figure 9) was presented as the fourth hit. This shows that the software is more powerful in distinguishing between different clones than the naked eye.

**Figure 13:**
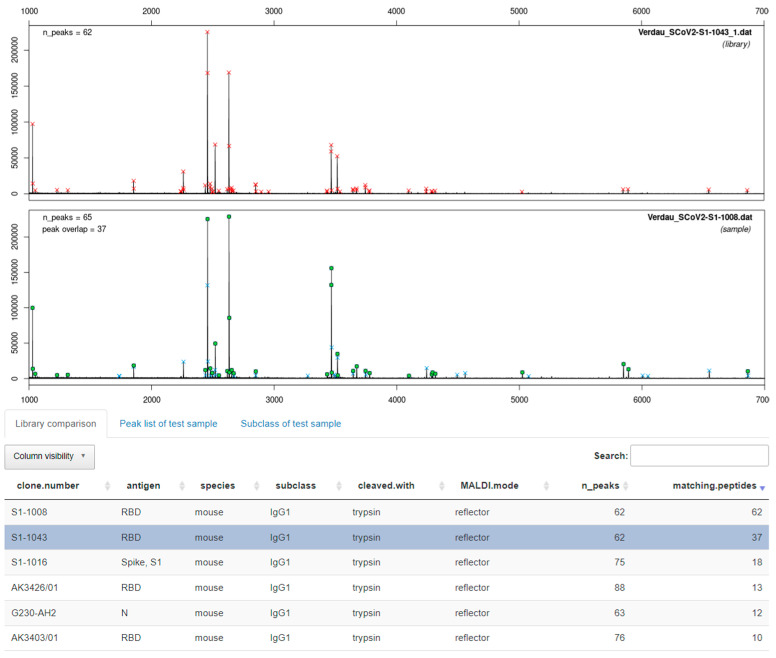
Screenshot of the graphical user interface of the ABID 2.0 software. The top spectrum is the library spectrum with the highest number of matching peaks with the sample spectrum (bottom spectrum). All library entries are shown in the bottom table and ranked by the number of matching peptides (last column). The software correctly identifies clone 1008 from the library as the highest match to the sample spectrum of clone 1043 with 37 matching peptides. The second-best match (S1-1016) to clone 1008 only has 18 matching peptides.

As a positive control, we show that ABID 2.0 is able to detect two clones that are indeed identical. In Figure 13, a screenshot is shown where the fingerprint of clone 1008 is matched against the default library containing the identical clone 1043. Both fingerprints were already compared in Figure 10. The results of the software are also conclusive. The best match is again redundantly given by the software to the identical fingerprint spectrum of clone 1008 also present in the library with 62 of 62 peptides matching. The real best match is shown in the second row (highlighted in blue). Thirty-seven matching peptides are given for clone 1043. It is expected that the spectral overlap is not 100% even though the clones are identical. The replicate measurement produced some small variance owing to the protein cleavage and MALDI measurement. However, the decisive criterion is a clear best match, which means a significant difference in matching peptides to the next library entry. Other fingerprints in the library have far fewer matching peptides with the fingerprint of clone 1008. Another example of a typical application of ABID is shown in Figure S8, where the clones 3396 and 3397 are compared.

Lastly, we demonstrated that based on the peptide mass fingerprints, ABID 2.0 could determine the antibody subclass. We added this functionality by implementing lists of subclass-specific peptide masses that are stored as separate library entries. These peptide masses were generated using in-silico-digested Fc domains from antibodies with known sequences and subclasses. Fingerprint spectra can then be compared to these subclass-specific peptide lists. Figure S9 compares these peptide lists with the fingerprint of clone 1254. Seven peptides were found that were also generated from in-silico digestions of other IgG1 antibodies. This suggests that clone 1254 has the subclass IgG1. Conventional subclass determination by sandwich ELISA discriminating between IgG1, IgG2a, IgG2b, and IgG3 shows the same result.

## 4. Discussion

### 4.1. Determination of Light Chain and Intact Mass

The determination of the intact mass of an antibody can be challenging as multiple glycoforms and other posttranslational modifications may be present, leading to considerable heterogeneity (Figure 1) [41,42]. Furthermore, without a high-resolution mass analyzer, the precision of the determination of the molecular mass is relatively poor, and hence this information might not be sufficient to discriminate between two different antibodies of similar mass (Figure 2) [43]. However, considering the short time needed for sample preparation and measurement (around 10 min), the determination of the intact mass may already be insightful. The light chain mass of an antibody (Figure 3) can be determined with a much lower uncertainty (Figure 4) due to the higher resolution of the mass spectrum at lower m/z values. In addition, the light chain usually does not contain any carbohydrates, and hence the resolution of glycoforms is not required. In combination with the intact mass, we were able to distinguish 21 of the 36 antibodies examined in this study (Figure 5, left). However, both the determination of the intact mass of an antibody and the mass of its light chain are not prerequisites for applying the peptide mass fingerprinting discussed below. All protocols can be used independently.

### 4.2. Peptide Mass Fingerprinting

Two novel peptide mass fingerprinting techniques were developed to distinguish a higher number of potentially identical clones. The key advantage over conventional protocols for peptide mass fingerprinting is the speedy experimental procedure. Both variants are one-pot reactions requiring low experimental effort and can be performed within 60 min.

The fingerprinting method based on the cleavage of diluted sulfuric acid (see Figure 6) is an improved version of our protocol for the identification of antibodies using diluted formic acid as the cleavage agent [25]. By substituting the reagent and increasing the temperature to 99 °C, we managed to shorten the reaction time from 5 h to just 30 min. In addition, the formation of unwanted byproducts by formylation can be avoided. Since the cleavage mechanism is known, the same protocol could also be used for the identification of any proteins via a comparison with in-silico-generated peptide lists. In that case, we expect the sequence coverage of smaller proteins to be even higher.

The fingerprinting method based on high-speed tryptic digestion (Figure 7) seems also to have never been reported before. Conventionally, tryptic digestion is carried out in several steps starting with the denaturation of the protein with chaotropic agents such as guanidinium-hydrochloride and reduction of disulfide bonds with dithiothreitol or TCEP. Then, alkylation of the cysteines is needed, followed by a buffer exchange before trypsin can be added. The incubation time for the tryptic digestion usually ranges from several hours to overnight [37]. In our protocol(s), the denaturation with heat (99 °C), TCEP, and omission of alkylation allow us to skip the buffer exchange by adding the trypsin after a short cooling period, making this a one-pot reaction finished in only 30 min. In the case of our sample set of antibodies, the information-rich fingerprint spectra were used to distinguish between two similar clones easily (Figure 8) without the need for any explicit sequence information.

Of 15 antibodies having overlapping confidence intervals with other antibodies (Figure 5 and Figure S1), two originated from different immunizations. It was shown that even though the two antibodies appear similar in intact and light chain mass and furthermore have the same subclass IgG1, they are indeed different based on the MALDI-TOF MS fingerprints obtained by chemical and enzymatic cleavages (Figure 9 or Figure 12).

Thirteen antibodies also had overlapping confidence intervals with at least one other antibody. However, all the respective clusters only contained antibodies originating from the same immunization. This is important because these clones may more likely have the same sequence. In the case of the clones 1008 and 1043, this was proven by sequencing of the hybridoma cells. The fingerprint spectra (Figure 10) suggest the same conclusion.

In many cases, it would be desirable to identify redundant clones as early as possible during hybridoma development. Usually, this information is only available after sequencing the hybridoma cells, which is still costly and time-consuming. However, together with relevant information obtained in a comprehensive screening approach for new hybridoma clones, e.g., on antibody affinity, specificity, or their epitope, a simple fingerprinting of the antibody done in less than an hour may help to determine which positive clones should be chosen for further development. Still, a few micrograms of antibody are needed for a fingerprint, which may not be available in a very early stage of the hybridoma process. In addition, the limiting factor of this approach may be interference by the cell culture medium, which would have to be removed by protein A/G purification.

### 4.3. Software-Assisted Antibody Identification with ABID 2.0

The software tool ABID 2.0 (Figure 11) was developed to automate the comparison of fingerprint spectra. This program has several advantages over its predecessor. The main one is an improved peak detection algorithm that allows the processing of isotopic resolved spectra. This makes it possible to use the reflector mode in the MALDI-TOF-MS instrument. We also used the reflector mode for our improved fingerprinting protocols described above. Furthermore, the higher resolution compared to the linear mode improves the matching algorithm as the allowed mass deviation between two peptides can be set to a much lower value. Several pre-processing options are now available to the user. For example, peak smoothing and baseline correction allow working on raw data directly. In addition, ABID 2.0 stores the library entries as simple mass lists and fingerprint spectra. This allows for an intuitive interface where the software results can be confirmed by visual inspection of the sample spectrum and the library spectra simultaneously. As the first step to antibody tracing, we implemented all the antibodies and their respective fingerprints as a default library in ABID 2.0. However, the library can be expanded with other antibodies if desired.

In Figure 12, an example is shown of a case where two antibodies appear to be visually similar based on their intact mass, light chain mass, and even their fingerprints, but the software correctly identifies them as two separate clones. The results are conclusive because the spectral overlap of the two clones is not significantly greater than the spectral overlap with other fingerprints in the library.

An example of the correct identification of identical clones is shown in Figure 13. There, the successful assignment of clone 1043 to clone 1008 in a database of 35 other clones confirms what was already expected after visual inspection of the two fingerprints. In this case, the difference between the best match to the second-best match is 19 peptides. This is usually enough to conclude the clone’s identity in a database of only a handful of clones or clones against the same antigen. However, it might be difficult to determine how great the difference in matching peptides to the next hit in the library needs to be to confirm a match. At some point, similar antibodies might give inconclusive results when it can be hard to tell the difference between identical and just very similar clones. Furthermore, with more fingerprints present in the library (e.g., several hundred), the difference in matching peptides from the second-best match might decrease simply by chance. Because of this lack of binary result output, a scientist must still examine the final result. In addition, it is relevant whether the antibodies are from the same immunization or not, as we showed earlier. Furthermore, it seems to be sensible to restrict the database to antibodies against relevant antigens.

Our results show that a library consisting of antibody fingerprints can be used to confirm the identity of a clone at a later stage without the need for sequence information. This approach may be helpful for researchers to independently check the identity of a clone sold by different vendors or distributors. In the future, publishing scientific work relying on one or several antibodies should always come with a fingerprint. This way, a database of antibody fingerprints used in publications can be set up.

Finally, the software tool may also determine the subclass of an antibody based on the similarities to other antibodies of the same subclass (Figure S8). Even though the algorithm is still at an early stage of development, the results are quite promising. The fingerprinting method might be a good alternative to lateral flow tests to determine the subclass of a new antibody quickly. However, this application needs some additional validation. In particular, more in-silico peptide masses per subclass need to be obtained to cover most possible polymorphisms in the Fc-domains of antibodies. Furthermore, the library might be extended to other species, such as rabbit or human antibodies.

## 5. Conclusions

Unambiguous identification of antibodies without knowledge of the amino acid sequence is challenging. Undoubtedly, the sequencing of diagnostic antibodies on the nucleic acid or protein level would be the gold standard in this respect. However, besides intellectual property issues, constraints in time and money remain severe limitations for these approaches. Due to these shortcomings, we developed three straightforward, fast, and inexpensive methods to identify monoclonal or recombinant antibodies. Thirty-five novel SARS-CoV-2 antibodies were examined in this study. The methods are based on the determination of the intact mass, light chain mass, and the generation of peptide mass fingerprints and can be finished in less than one hour with minimal experimental effort.

We could show that in many cases, the determination of intact and light chain masses, even without a high-resolution mass analyzer, is already sufficient to prove the non-identity of two clones. Nevertheless, more powerful methods are needed for the successful distinction of dozens or even hundreds of clones. In this work, two novel peptide mass fingerprinting protocols are presented that provide a combined sequence coverage of more than 80% in less than one hour. A comparison of differing fingerprints successfully demonstrated their effectiveness in distinguishing different clones. In contrast, it was also shown that the fingerprints of sister clones with a complete sequence identity could be reproduced, which means that the fingerprints produce a unique, sequence-related characteristic for each antibody. Because a visual comparison of fingerprints is challenging and tedious with a high number of clones, the web-based software ABID 2.0 was developed. With its optimized peak detection algorithm, ABID 2.0 allows the user to compare a single fingerprint spectrum with a whole library of fingerprints of other antibodies. In this work, we exemplarily show that the software correctly identifies any clone in question already present in the library. It is important to note that false-positive identifications might increase with a rising number of antibodies in the library. For this reason, we included filter options in the software that make it possible to compare antibodies based on certain criteria, such as antigen or subclass. Therefore, we expect that the traceability of antibodies in the scientific literature will be improved considerably. Using these fingerprinting methods in combination with a tool such as ABID 2.0, access to sequence information is unnecessary for antibody identification. Ultimately, software such as ABID 2.0 could be used to link unique and sequence-independent fingerprints to other antibody identifiers [44,45], such as Research Resource Identifiers (RRIDs) [46] or entries in CiteAb [47]. Finally, we present a proof of concept that the subclass determination of antibodies is also feasible.

## Figures and Tables

**Table 1:** Murine SARS-CoV-2 antibodies used in this study.

Clone	Abbreviation	Isotype	Concentration (g/L)	Antigen
S1-556	556	IgG1	1.84	S1
S1-1008	1008	IgG1	1.81	RBD
S1-1016	1016	IgG1	1.58	S1
S1-1043	1043	IgG1	1.74	RBD
S1-1047	1047	IgG1	1.91	S1
S1-1058	1058	IgG2a	1.65	S1
S2-1254	1254	IgG1	1.84	S2
S2-2843	2843	IgG1	1.81	S2
S1-3332	3332	IgG1	1.58	S1
S1-3355	3355	IgG1	1.77	RBD
S2-3389	3389	IgG1	1.75	S2
AK3396/01	3396	IgG2b	1.00	RBD
AK3397/01	3397	IgG2b	1.40	RBD
AK3398/02	3398	IgG1	1.50	RBD
AK3400/01	3400	IgG2b	1.10	RBD
AK3402/01	3402	IgG2b	1.15	RBD
AK3403/01	3403	IgG1	0.70	RBD
AK3404/02	3404	IgG1	1.45	RBD
AK3405/01	3405	IgG1	0.75	RBD
AK3421/01	3421	IgG1	2.35	S1
AK3422/01	3422	IgG2a	2.05	S1
AK3423/01	3423	IgG1	1.15	S1
AK3424/01	3424	IgG2a	1.25	S1
AK3425/01	3425	IgG1	0.95	S1
AK3426/01	3426	IgG1	1.00	RBD
AK3427/01	3427	IgG1	1.15	RBD
AK3428/01	3428	IgG1	1.05	S1
AK3429/01	3429	IgG1	1.30	RBD
AK3439/01	3439	IgG1	1.25	S1
G230-AH2	AH2	IgG1	2.00	N
G230-HC9	HC9	IgG2a	1.05	N
G230-DE6	DE6	IgG2b	2.10	N
G230-GG4	GG4	IgG1	3.10	N
G230-JC3	JC3	IgG2b	1.75	N
G229-FA10	FA10	IgG1	0.75	N

Color code: blue—Robert Koch Institute; green—InVivo/Bruker; grey—Hybrotec/UP-Transfer.

## Data Availability

The data presented in this study are available in supplementary materials and on Zenodo https://doi.org/10.5281/zenodo.6375803.

## Supplementary Materials

The following are available online at https://www.mdpi.com/article/10.3390/antib11020027/s1; Figure S1: Light chain and intact masses of antibodies with overlapping mass ranges relative to the NIST antibody; Figure S2: Acidic cleavage: MALDI-TOF fingerprint spectrum of NIST-mAb 8671 after 30 min incubation with diluted sulfuric acid and TCEP at 99 °C; Figure S3: Peptide mass fingerprint of NIST antibody (mAb 8671) was obtained from cleavage with 2% formic acid (S/N 16.0), 5 mM sulfuric acid (S/N 39.3), and 20 mM phosphoric acid (S/N 15.9); Figure S4: MALDI-TOF MS fingerprints of NIST antibody 8671 generated with different MALDI matrices: 2,5 DHAP (a), CHCA (b), DHB with an automated random pathway of the laser (c), and DHB with manual laser control (d); Figure S5: Tryptic digest: MALDI-TOF fingerprint spectrum of NIST-mAb 8671 after 15 min of incubation with TCEP at 99 °C, followed by 15 min of incubation with trypsin at 55 °C.; Figure S6: Tryptic digests of NIST-mab 8671 with and without the addition of an alkylation step; Figure S7: The combined sequence coverage of NIST antibody mAb 8671; Figure S8: Comparison by ABID 2.0 software: The first spectrum is the library spectrum with the highest number of matching peaks with the sample spectrum (second spectrum); Figure S9: Screenshot of the software tool ABID 2.0. The tool compares peptide mass lists from in-silico digested Fc domains from antibodies with known sequences and subclasses.
