# Supplementary material for: MALDI-TOF-MS-Based Identification of Monoclonal Murine Anti-SARS-CoV-2 Antibodies within One Hour
Source: Antibodies (Basel). 2022 Apr 14;11(2):27. doi: 10.3390/antib11020027 (PMC9036215; doi:10.3390/antib11020027)
Supplement: Supplementary file 1 [file antibodies-11-00027-s001.zip › antibodies-1659003-supplementary.pdf]

antibodies

MDPI

/Supplementary materials

MALDI-TOF-MS-based identification of monoclonal murine anti-SARS-CoV-2 antibodies within one hour

Georg Tscheuschner [1], Melanie N. Kaiser [1], Jan Lisec [1], Denis Beslic [2], Thilo Muth [1], Maren Krüger [2], Hans Werner Mages [2], Brigitte G. Dorner [2], Julia Knospe [3], Jörg A. Schenk [4,5], Frank Sellrie [4,5] and Michael G. Weller [1,*]

1 Federal Institute for Materials Research and Testing (BAM), Richard-Willstätter-Strasse 11, 12489 Berlin, Germany; georg.tscheuschner@bam.de (G.T.); melliehco@hotmail.de (M.N.K.); jan.lisec@bam.de (J.L.); thilo.muth@bam.de (T.M.); michael.weller@bam.de (M.W.)
2 Robert Koch Institute (RKI), Seestraße 10, 13353 Berlin, Germany; kruegerm@rki.de (M.K.); Magesh@rki.de (H.W.M.) dornerb@rki.de (B.G), beslicd@rki.de (D.B.)
3 InVivo BioTech Services GmbH/Bruker, Neuendorfstraße 24a, 16761 Hennigsdorf, Germany; julia.knospe@bruker.com (J.K.)
4 HybroTec GmbH, Am Mühlenberg 11, 14476 Potsdam, Germany; joerg.schenk@hybrotec.com (J.A.S.)
5 UP Transfer GmbH an der Universität Potsdam, Am Neuen Palais 10, 14469 Potsdam, Germany; frank.sellrie@up-transfer.de (F.S.)
* Correspondence: michael.weller@bam.de; Tel.: +49-30-8104-1150

Determination of intact and light chain masses (IgG)

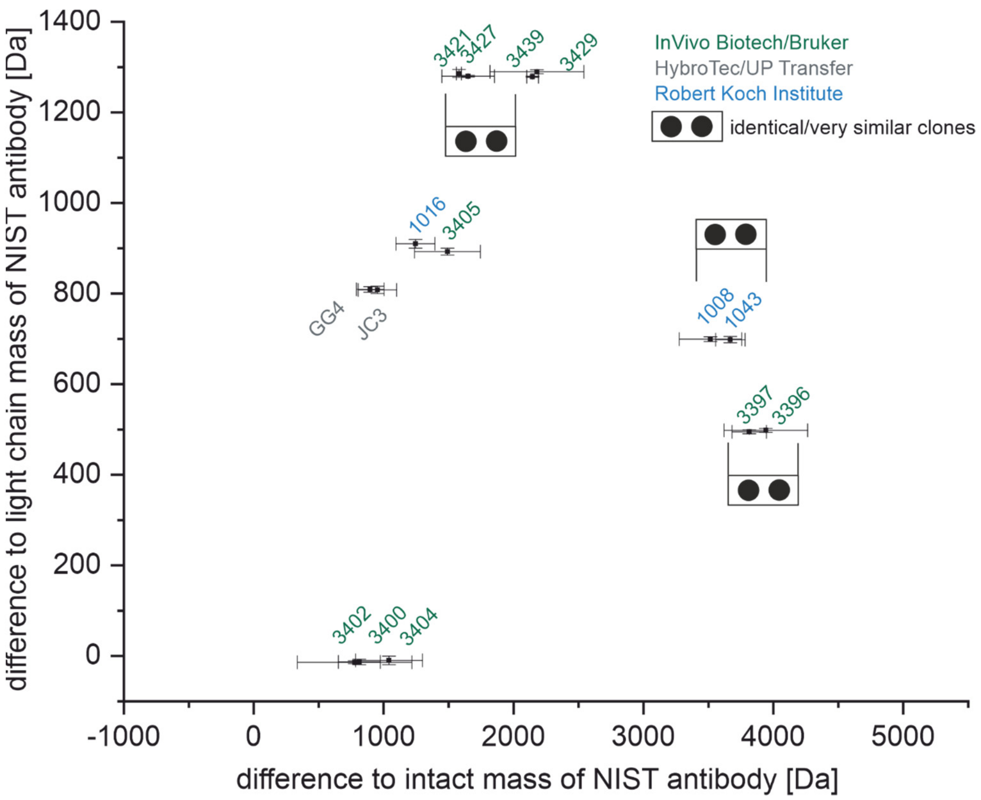

Figure S1. Light chain and intact masses of antibodies with overlapping mass ranges relative to the NIST antibody.

Sequence coverage of peptide mass fingerprinting by partial acidic hydrolysis

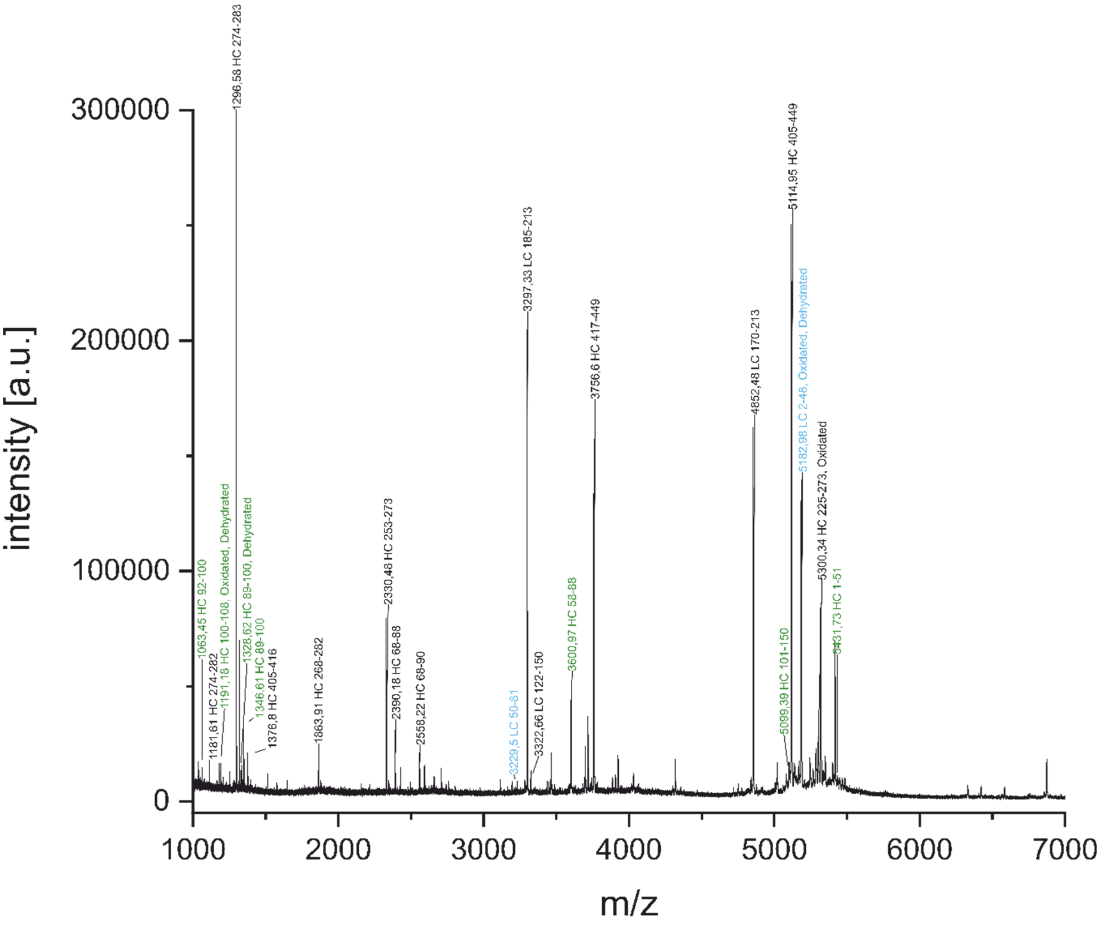

Figure S2. Acidic cleavage: MALDI-TOF fingerprint spectrum of NIST-mAb 8671 after 30 minutes incubation with diluted sulfuric acid and TCEP at 99 °C. Peptide peaks were assigned to the NIST sequence. Colored annotations mark peptides derived from CDR regions, green for the heavy chain (HC) and blue for the light chain (LC). The sequence coverage is around 61%.

Method optimization: Peptide mass fingerprinting with different acids as cleavage agents

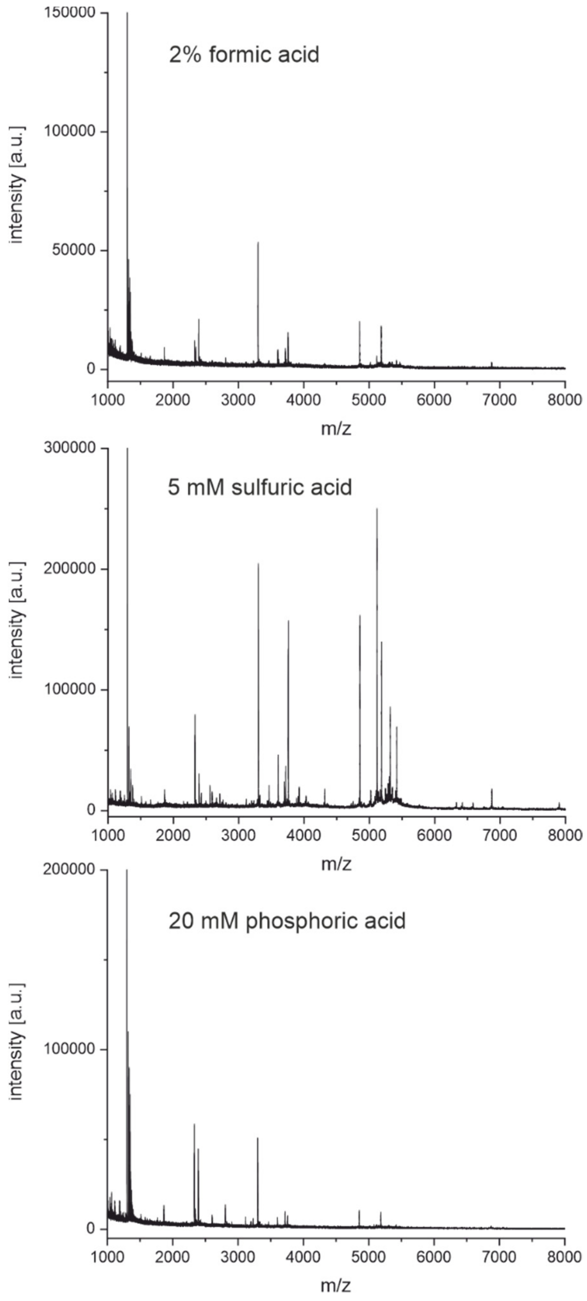

Figure S3. Peptide mass fingerprint of NIST antibody (mAb 8671) was obtained from cleavage with 2% formic acid (S/N 16.0), 5 mM sulfuric acid (S/N 39.3), and 20 mM phosphoric acid (S/N 15.9). The signal-to-noise ratios (S/N) were calculated from the signal intensity of the monoisotopic peak at 3297.59 Da divided by the difference of maximum and minimum intensity between 3308 Da and 3316 Da.

Many diluted acids may be applied to selectively cleave at aspartic acid residues and generate fingerprints in a short time. However, as Figure S3 shows, 5 mM sulfuric acid produces the best results with a high number of peptide peaks and a good signal-to-noise ratio (S/N 39.3). Interestingly, the relative peak intensities vary greatly between the spectra, even if the peptide masses are the same. We, therefore, recommend using 5 mM sulfuric acid as a cleavage reagent to generate the fingerprints. Unwanted peptide modification, for example, formylation, is also not an issue with this cleavage reagent compared to formic acid.

Method optimization: MALDI matrix

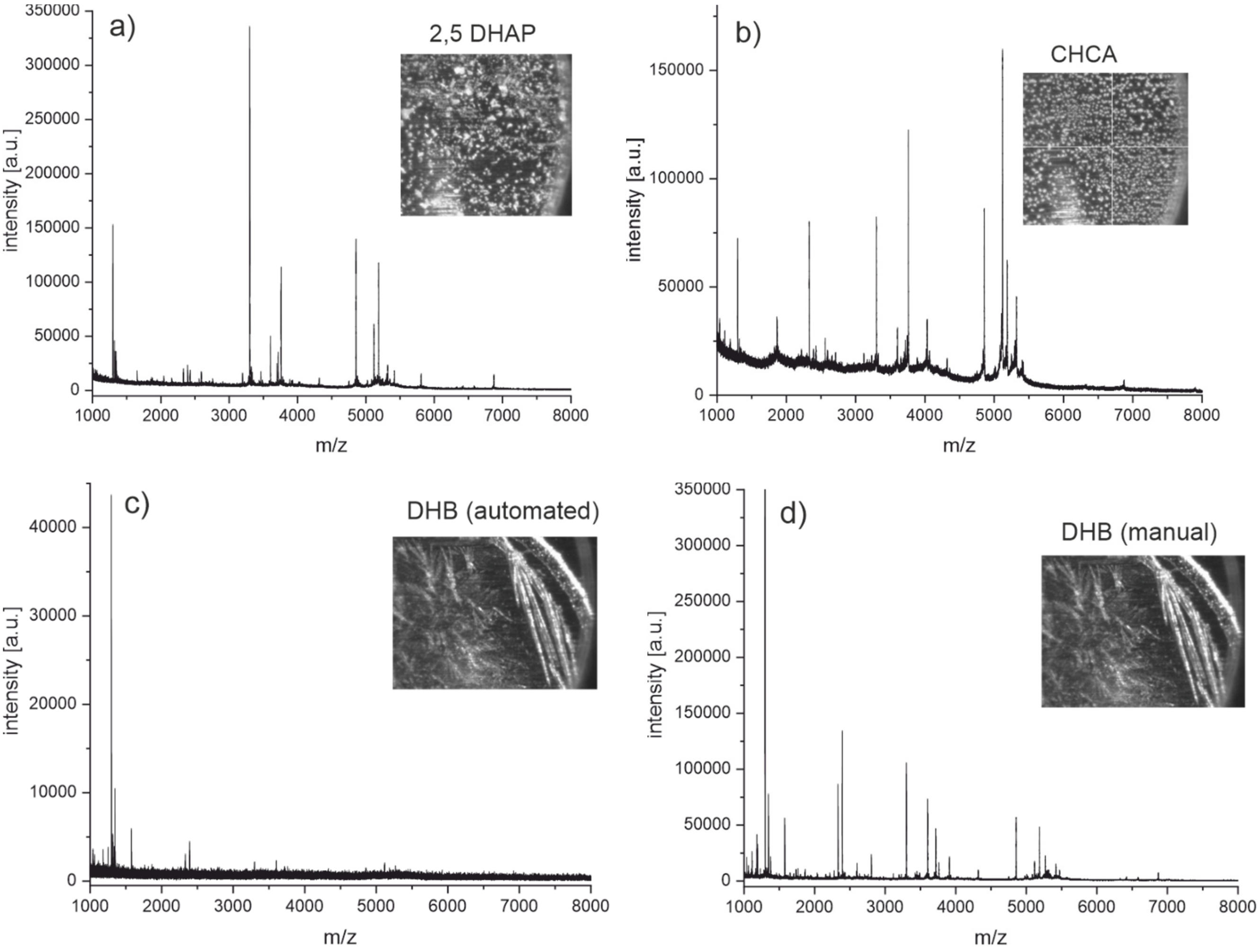

Figure S4. MALDI-TOF MS fingerprints of NIST antibody 8671 generated with different MALDI matrices: 2,5 DHAP (a), CHCA (b), DHB with an automated random pathway of the laser (c), and DHB with manual laser control (d).

The MALDI matrix seems to play a critical role in the generation of information-rich fingerprint spectra (Figure S4). The fingerprints seem to produce the same peptides but with varying relative intensities. Furthermore, 2,5-DHAP (a) and DHB (with manual laser control, d) produce spectra with more peptides and better signal-to-noise ratios than CHCA (b). However, the spectrum generated with DHB with an automated random pathway of the laser (c) is poor, even though it was acquired on the same sample spot. The sweet-spot focusing can explain the differences between manual laser control and the automated one. This way, the large crystals from the DHB matrix are targeted, while the automated software was not able to perform a sweet-spot focusing. To facilitate automation of the workflow, we, therefore, recommend using 2,5-DHAP as the MALDI matrix. This matrix compound crystallizes homogenously, which facilitates automation during spectra acquisition.

Sequence coverage of peptide mass fingerprinting by tryptic digestion

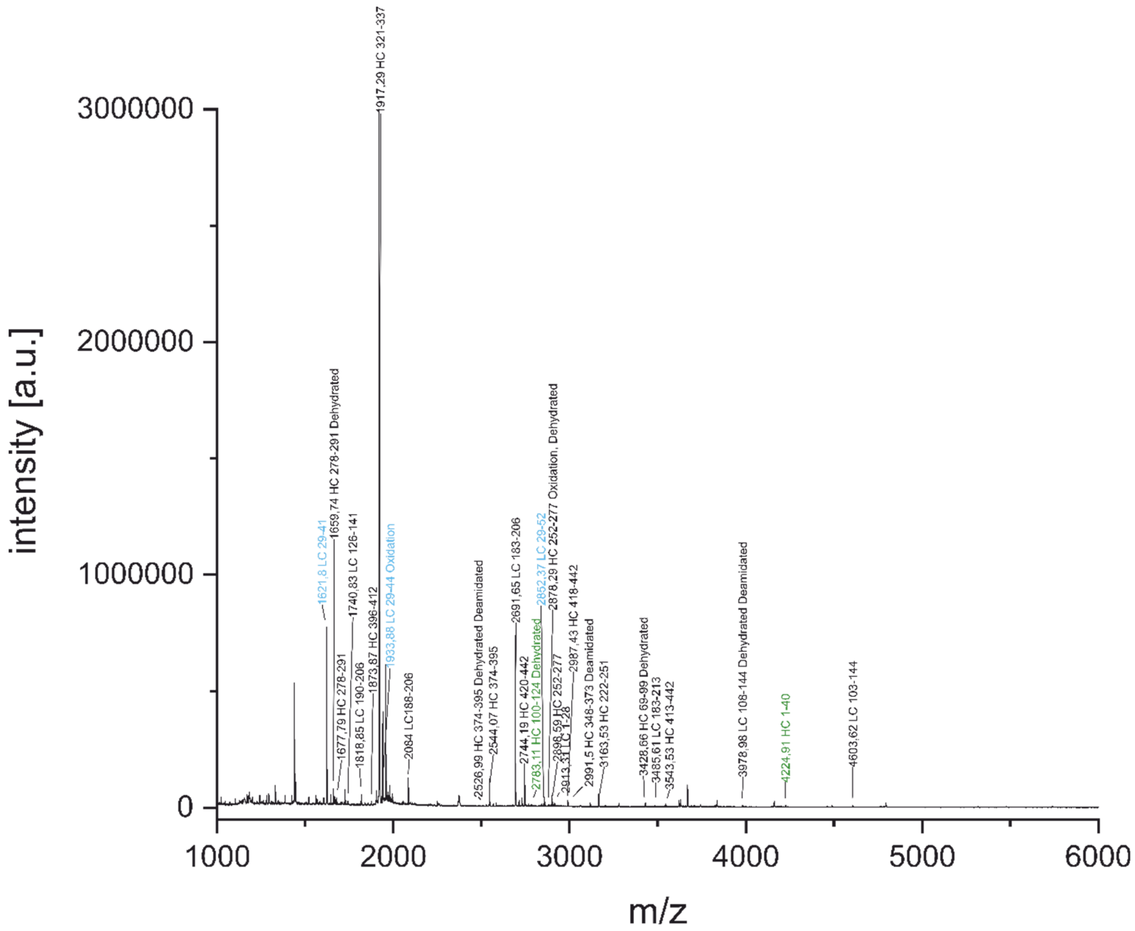

Figure S5. Tryptic digest: MALDI-TOF fingerprint spectrum of NIST-mAb 8671 after 15 minutes of incubation with TCEP at 99 °C, followed by 15 minutes of incubation with trypsin at 55 °C. Peptide peaks are assigned to the NIST sequence. Colored peaks mark peptides derived from CDR regions, green for the heavy chain (HC) and blue for the light chain (LC). The sequence coverage is around 65%.

The addition of an alkylation step did not improve the sequence coverage. In Figure S6, a comparison is shown between two fingerprints. The top fingerprint was generated by adding an alkylation step at room temperature after the denaturation of the antibody at 99 °C. The bottom fingerprint was generated by incubation of the antibody at room temperature after the denaturation at 99 °C. For both fingerprints, the sequence coverage is around 50%. This indicates that the alkylation of peptides in this context does not improve the coverage. In fact, the extra incubation time at room temperature (with or without an alkylating agent) even leads to a loss of coverage, likely caused by the refolding of the protein. Hence, alkylation should not be used in this protocol. The rapid addition of trypsin at 55 °C after the denaturing step might be crucial in order to conserve most of the denatured protein for the remainder of the 15 minutes digestion.

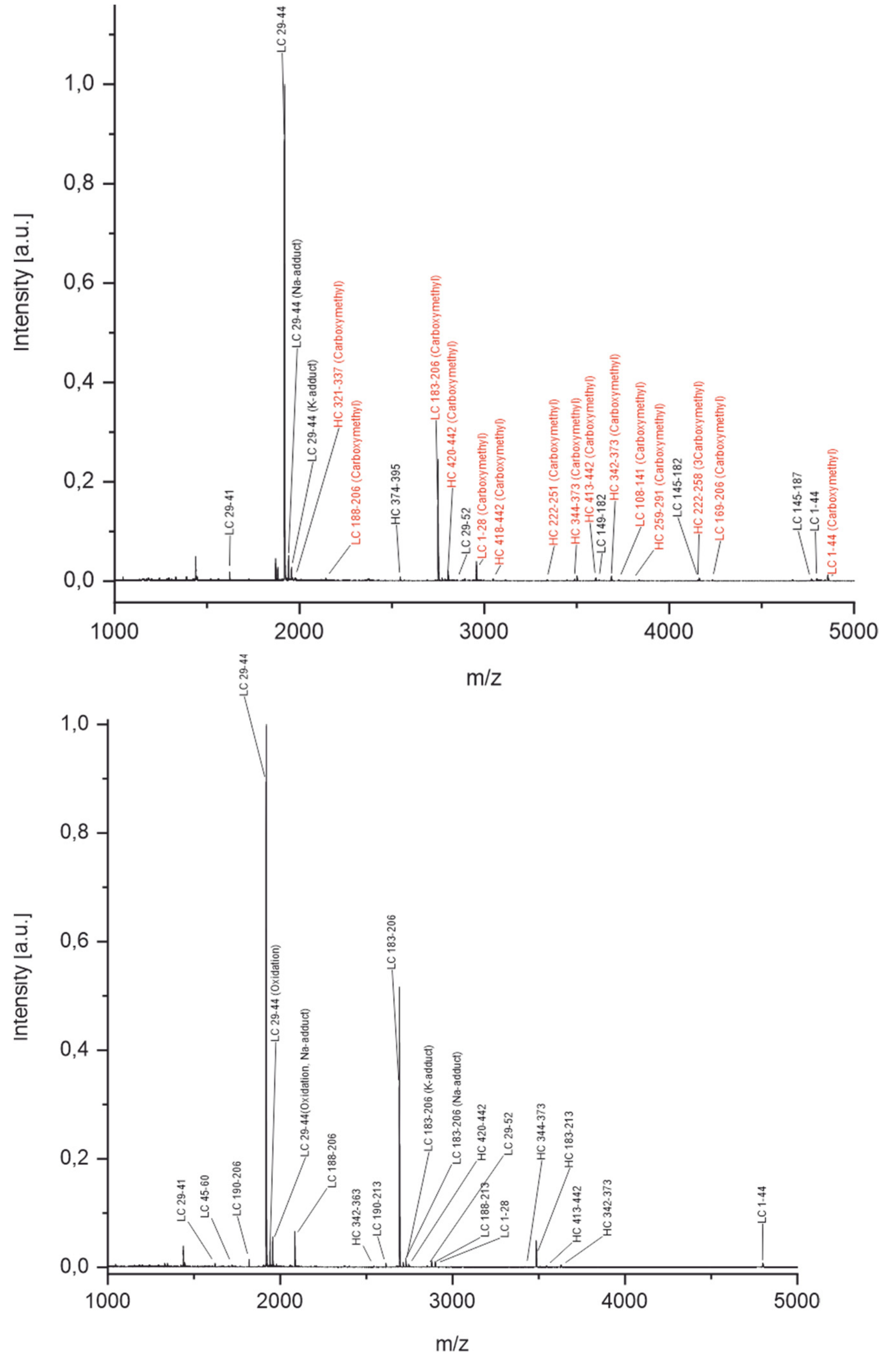

Figure S6. Tryptic digests of NIST-mab 8671 with and without the addition of an alkylation step.

Top spectrum: The antibody was incubated in 0.1 M tris-buffer (pH 7.8) with TCEP at 99 °C for 15 minutes. After the sample had cooled down to room temperature, a final concentration of iodoacetic acid (IAA) of 6 mM was added, and the sample was incubated for another 30 minutes at room temperature. Next, the temperature was raised to 55 °C, and trypsin was added. Digestion was allowed to proceed for 15 minutes. The sequence coverage was 49%. Bottom spectrum: The antibody was incubated in 0.1 M tris-buffer (pH 7.8) with TCEP at 99 °C for 15 minutes. Afterward, it was incubated for another 30 minutes at room temperature. Next, the temperature was raised to 55 °C, and trypsin was added. Digestion was allowed to proceed for 15 minutes. The sequence coverage was 50%. Peptide peaks are assigned to the NIST-mAb 8671 sequence. Peaks colored red are derived from carboxymethylated cysteine-containing peptides.

Heavy Chain of NIST-mAb 8671

QVTLRESGPA LVKPTQTLTL TCTFSGFSLS **TAGMSVG**WIR QPPGKALEWL A**DIWWDDKKH YNPSLKD**RLT IS-KDTSKNQV VLKVTNMDPA DTATYYCAR**D MIFNFYFDV**W GQGTTVTVSS ASTKGPSVFP LAPSSKSTSG GTAALGCLVK DYFPEPVTVS WNSGALTSGV HTFPAVLQSS GLYSLSSVVT VPSSSLGTQT YICNVNHKPS NTKVDKRVEP KSCDKTHTCP PCPAPELLGG PSVFLFPPKP KDTLMISRTP EVTCVVVDVS HEDPEVKFNW YVDGVEVHNA KTKPREEQYN STYRVVSVLT VLHQDWLNGK EYKCKVSNKA LPAPIEKTIS KAKGQPREPQ VYTLPPSREE MTKNQVSLTC LVKGFYPSDI AVEWESNGQP ENNYKTTPPV LDSDGSFFLY SKLTVDKSRW QQGNVFSCSV MHEALHNHYT QKSLSLSPG

Light Chain of NIST-mAb 8671

DIQMTQSPST LSASVGDRVT ITC**SASSRVG YMH**WYQQKPG KAPKLLIY**DT SKLAS**GVPSR FSGSGSGTEF TLTISSLQPD DFATYYC**FQG SGYPFT**FGGG TKVEIKRTVA APSVFIFPPS DEQLKSGTAS VVCLLNNFYP REAK-VQWKVD NALQSGNSQE SVTEQDSKDS TYSLSSTLTL SKADYEKHKV YACEVTHQGL SSPVTKSFNR GEC

Figure S7. The combined sequence coverage of NIST antibody mAb 8671. Amino acids marked yellow are covered in the fingerprint spectrum generated with diluted sulfuric acid. Amino acids underlined red are covered in the fingerprint spectrum generated with trypsin. Amino acids with bold letters are from **CDR** regions. The combined sequence coverage is around 82%.

Software tool ABID 2.0

In the following, a typical application of ABID 2.0 is shown, where supposed duplicates of the same clone can be detected in a library of dozens of clones (Figure S8). Both clones, 3396 and 3397, seem to be rather similar by visual examination.

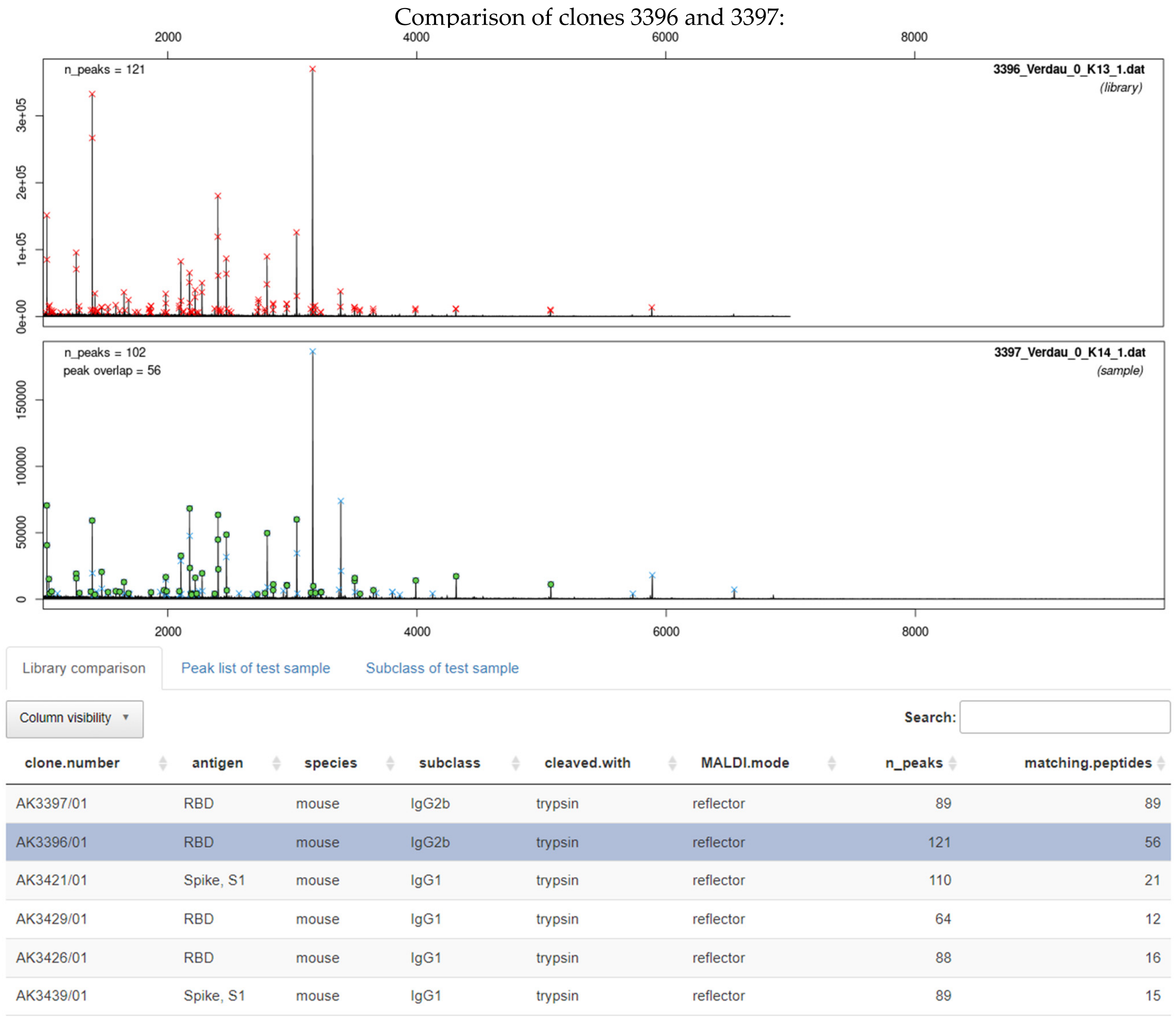

clone.number	antigen	species	subclass	cleaved.with	MALDI.mode	n_peaks	matching.peptides
AK3397/01	RBD	mouse	IgG2b	trypsin	reflector	89	89
AK3396/01	RBD	mouse	IgG2b	trypsin	reflector	121	56
AK3421/01	Spike, S1	mouse	IgG1	trypsin	reflector	110	21
AK3429/01	RBD	mouse	IgG1	trypsin	reflector	64	12
AK3426/01	RBD	mouse	IgG1	trypsin	reflector	88	16
AK3439/01	Spike, S1	mouse	IgG1	trypsin	reflector	89	15

Figure S8. Comparison by ABID 2.0 software: The first spectrum is the library spectrum with the highest number of matching peaks with the sample spectrum (second spectrum).

All library entries are shown in the table and ranked by the number of matching peptides (last column). The first line shows the comparison of the spectrum with itself, which leads to the result that all peaks match. The second line (gray bar) determines clone 3396 from the library as the highest match to the sample spectrum of clone 3397 with 56 matching peptides. The third line shows the second-best match (AK3421/01, third row) to clone 3397, which has only 21 matching peptides. The significant difference between the best hit and the second one is crucial for a decision. Based on their overlapping intact masses and light chain masses, fingerprints, subclasses, and antigens, we expect 3396 and 3397 to be very similar, if not identical.

Another feature of ABID 2.0 is the determination of subclasses from the antibody fingerprint spectrum (Figure S9). This works because the subclass information is inherent to the m/z values of Fc-specific peptides in the mass spectrum. ABID 2.0 was fed peptide

mass lists from in-silico digested Fc domains from antibodies with known sequences and subclasses. The algorithm then compares these lists with the sample spectrum to determine the best match. In Figure S11 the fingerprint spectrum of clone 1254 is loaded into the software. ABID 2.0 determines IgG1 as the subclass since the fingerprint spectrum has 7 peptide peaks that have the same masses as other IgG1 specific peptides. Conventional subclass determination shows the same result for clone 1254.

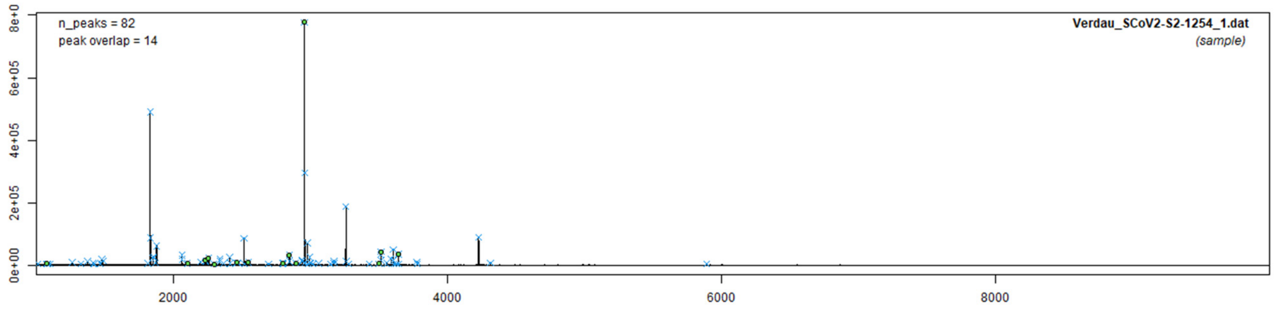

Library comparison | Peak list of test sample | Subclass of test sample

Show 10 entries

Search: t

Organism	Class	Digestion	n_peaks	n_overlap
mouse	IgG1	T	84	7
mouse	IgG2A	T	87	2
mouse	IgG2B	T	109	2
mouse	IgG3	T	99	2
mouse	IgA	T	49	0

Figure S9. Screenshot of the software tool ABID 2.0. The tool compares peptide mass lists from in-silico digested Fc domains from antibodies with known sequences and subclasses.

The fingerprint spectrum of clone 1254 contains 7 peptides that are also found in other antibodies with the subclass IgG1. Conventional subclass determination by sandwich ELISA discriminating between IgG1, IgG2a, IgG2b, and IgG3 shows the same result (data not shown).

MALDI-TOF-MS spectra of all clones (acidic and tryptic cleavage)

Raw data are accessible on Zenodo: https://doi.org/10.5281/zenodo.6375803

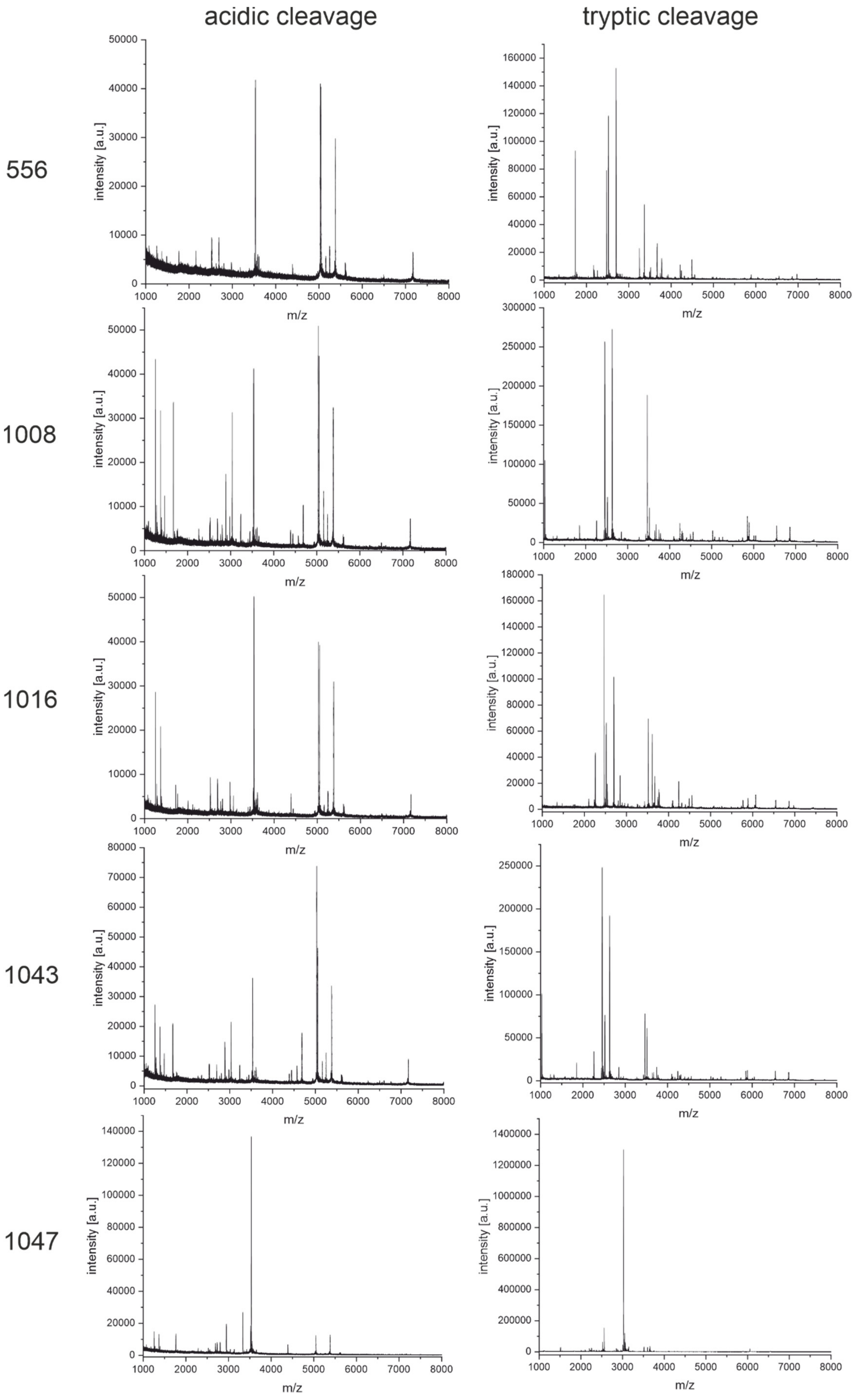
acidic cleavage
tryptic cleavage
556
1008
1016
1043
1047
intensity [a.u.]
m/z

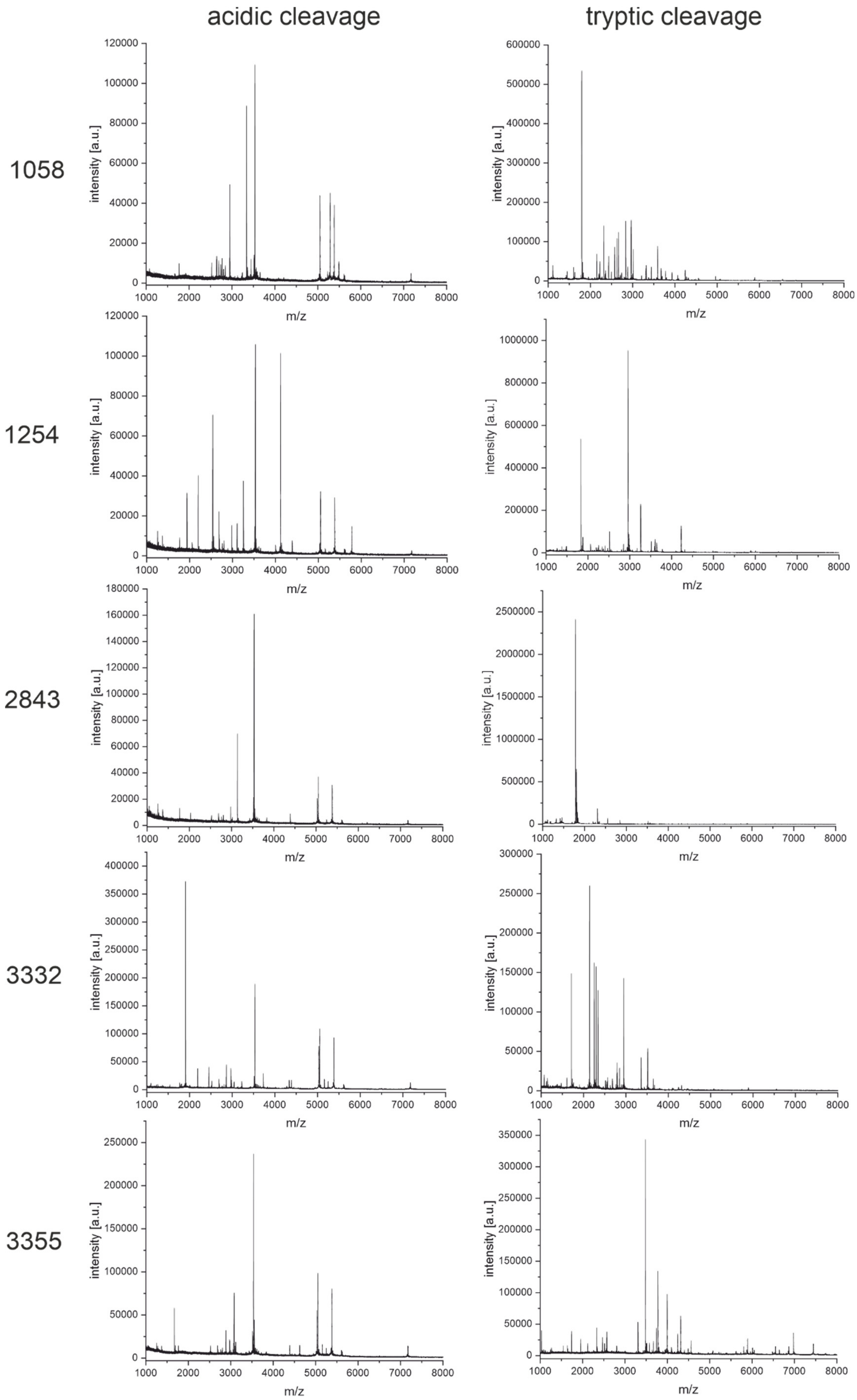
acidic cleavage
tryptic cleavage
1058
1254
2843
3332
3355
intensity [a.u.]
m/z

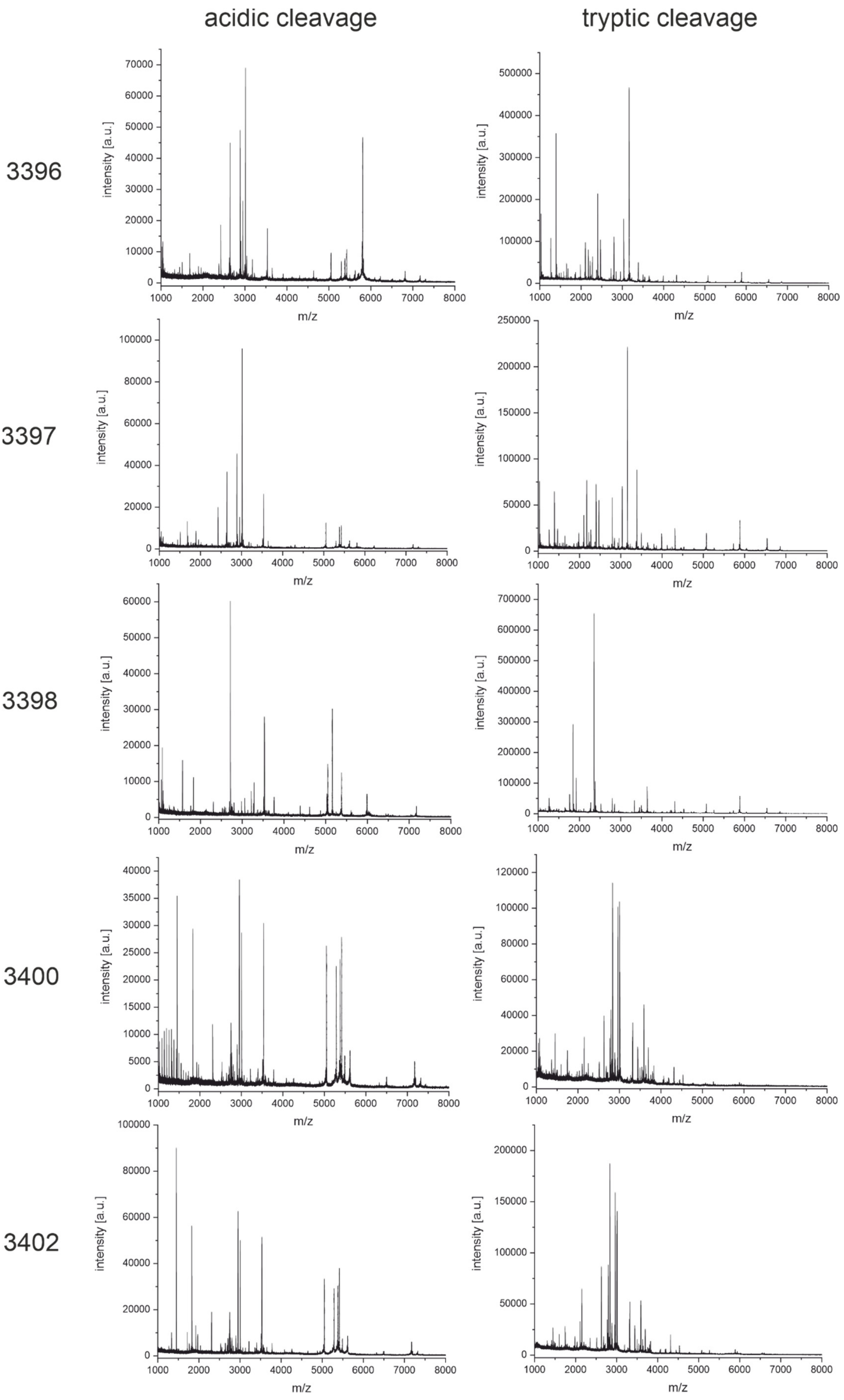
acidic cleavage
tryptic cleavage
3396
3397
3398
3400
3402
intensity [a.u.]
m/z

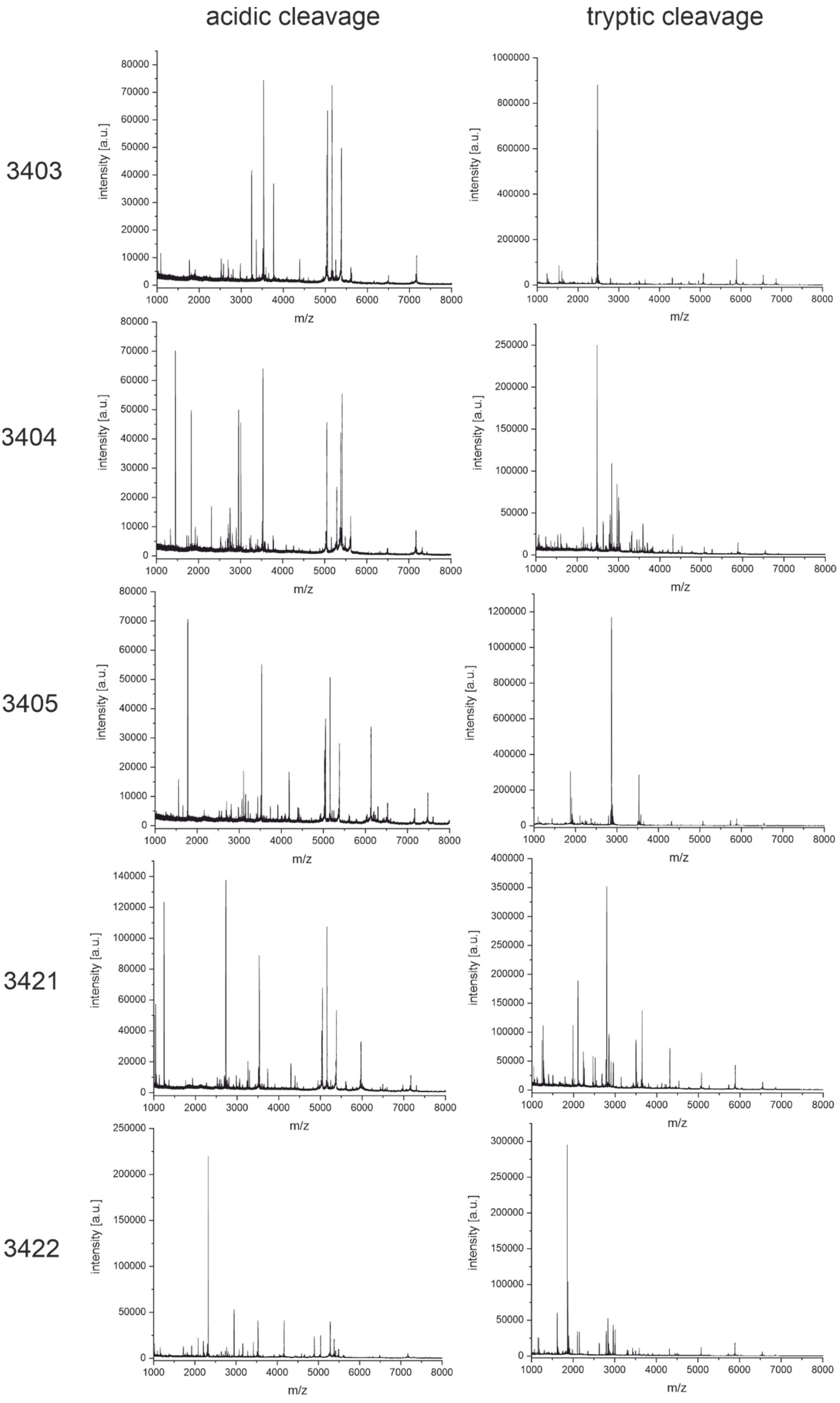
acidic cleavage
tryptic cleavage
3403
3404
3405
3421
3422
intensity [a.u.]
m/z

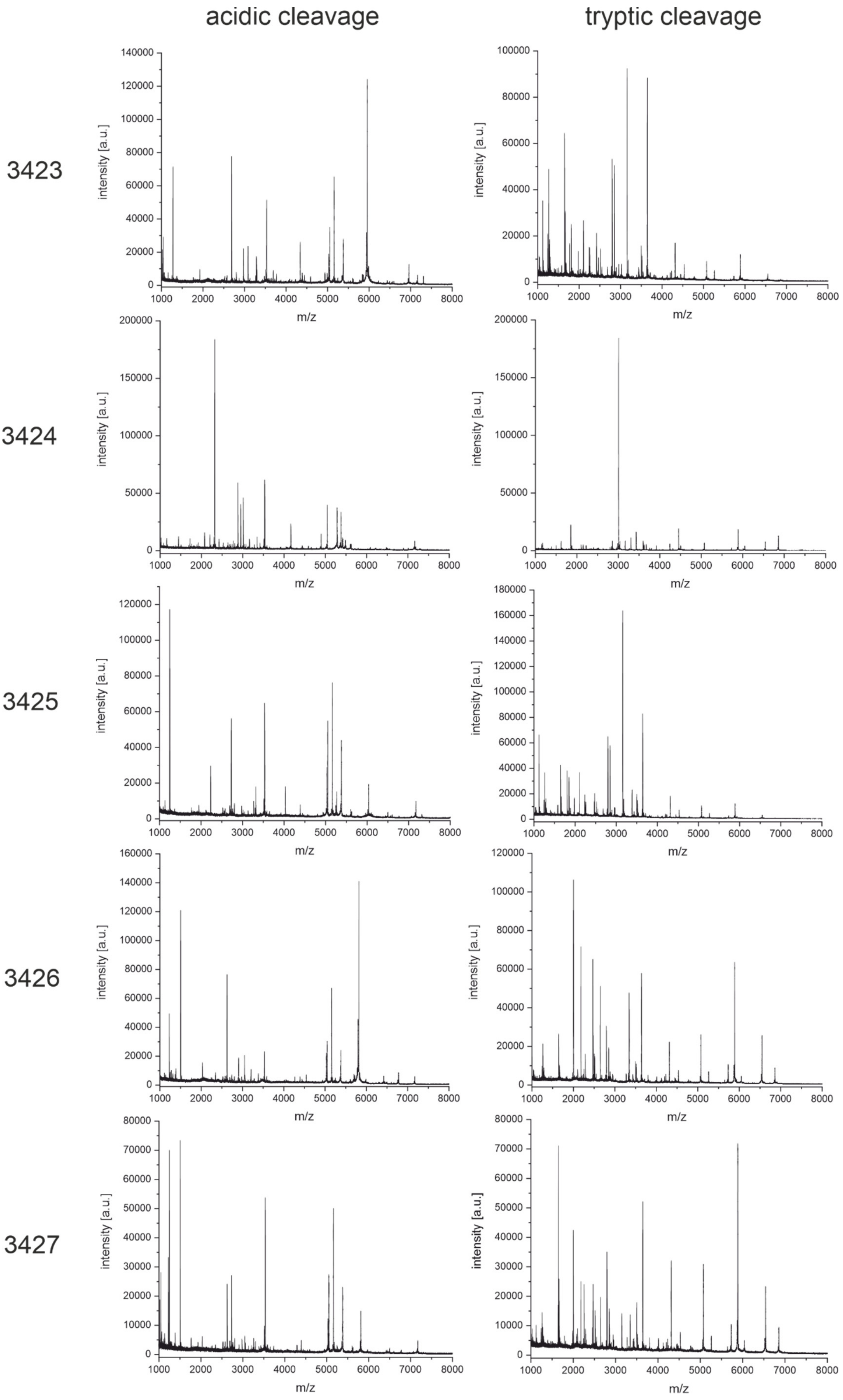
acidic cleavage
tryptic cleavage
3423
3424
3425
3426
3427
intensity [a.u.]
m/z

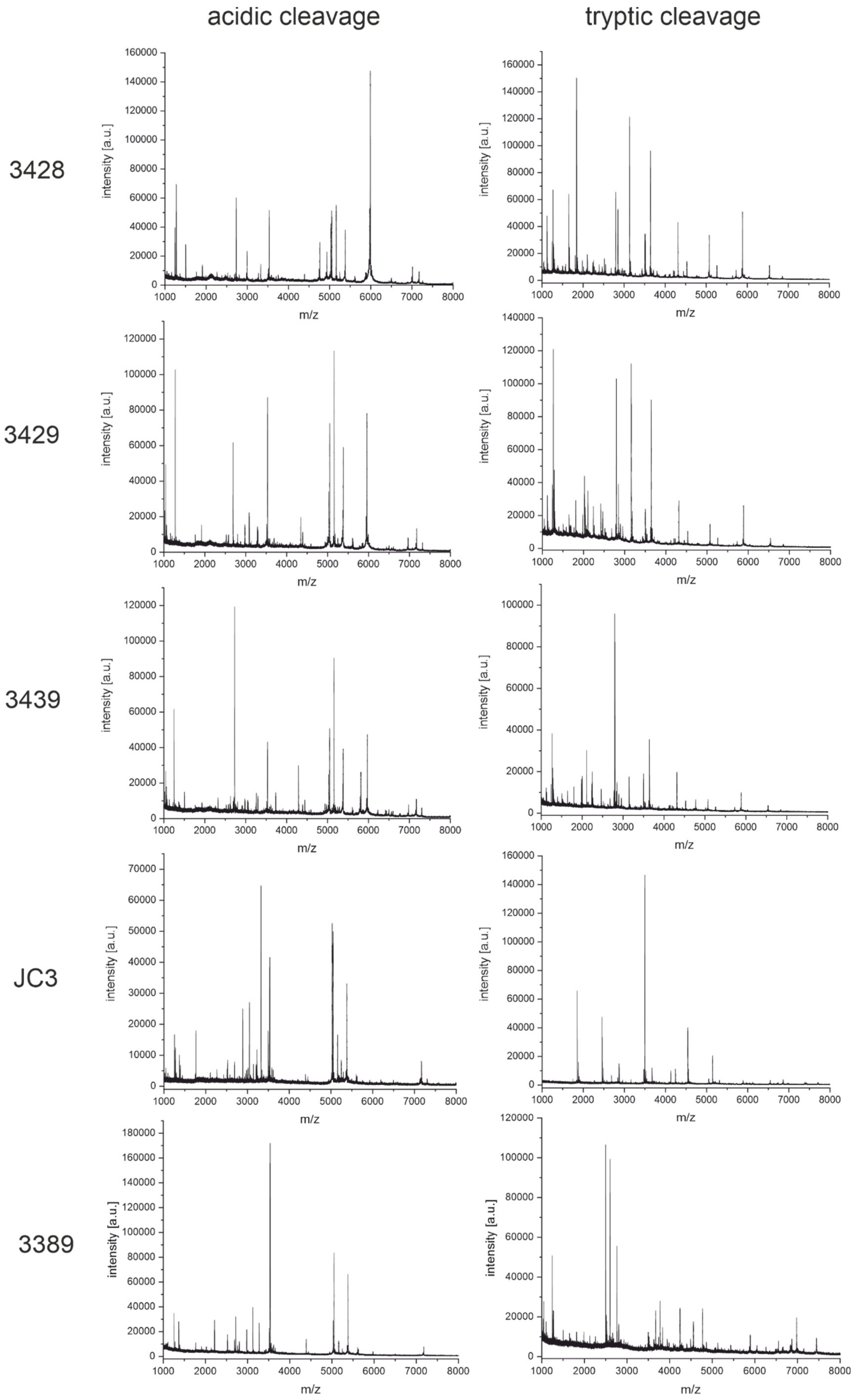
acidic cleavage
tryptic cleavage
3428
3429
3439
JC3
3389
intensity [a.u.]
m/z

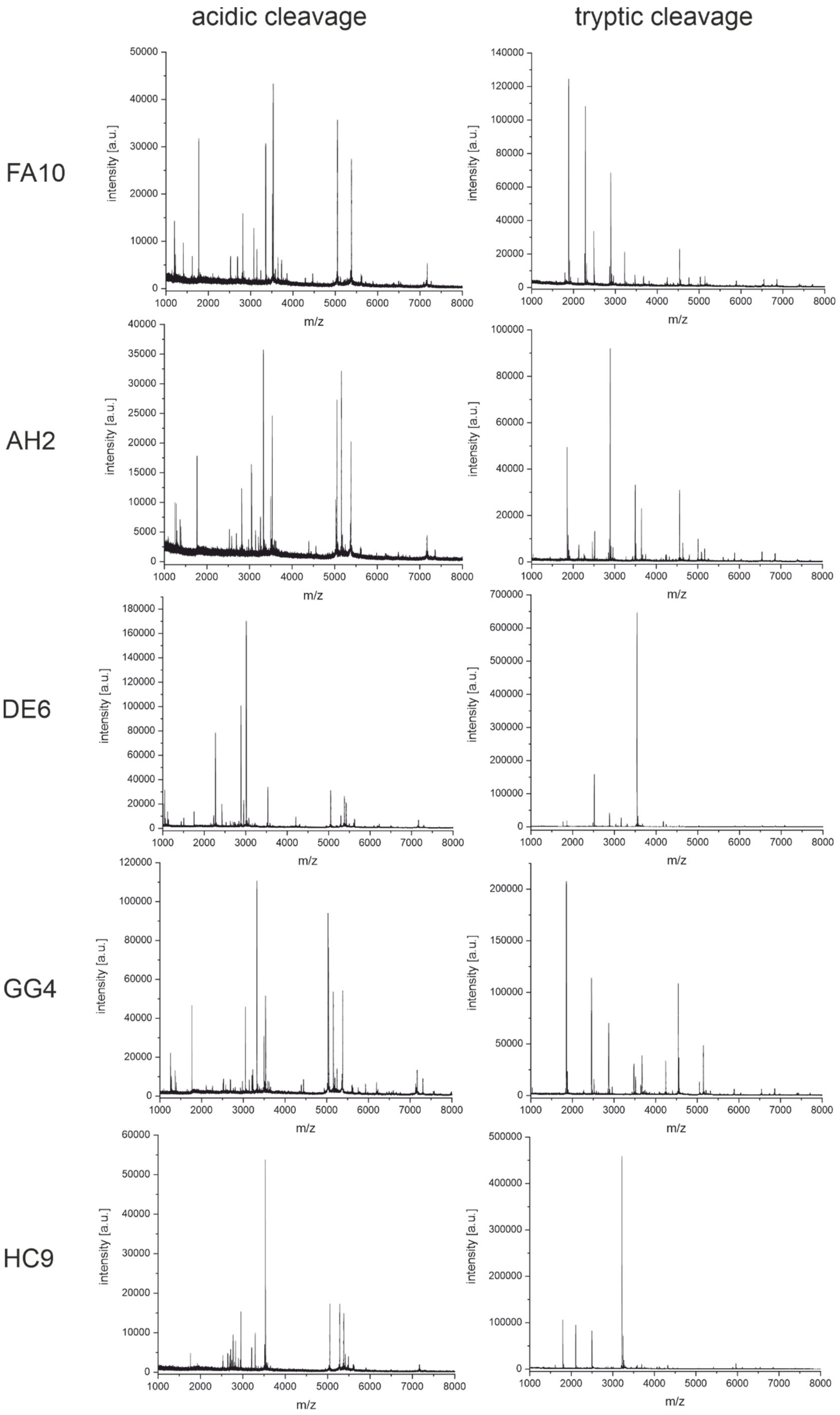
acidic cleavage
tryptic cleavage
FA10
AH2
DE6
GG4
HC9
intensity [a.u.]
m/z
